# Environmental DNA sampling workflow matters: testing different approaches on marine microbial assemblages in three coastal areas of Greece

**DOI:** 10.3389/fmicb.2026.1953819

**Published:** 2026-09-25

**Authors:** Alexandra Zachariadou, Dionysios E. Raitsos, Alexandra Meziti, Chrysoula Gubili, Panagiota Xanthopoulou, Natassa Stefanidou, Konstantinos Proios, Michalis Omirou, Johana Rodosthenous, Christiana Hadjimichael, Antonios Mazaris, Savvas Genitsaris

**Affiliations:** 1 Section of Ecology & Taxonomy, Department of Biology, National and Kapodistrian University of Athens, Athens, Greece; 2 Section of Zoology - Marine Biology, Department of Biology, National and Kapodistrian University of Athens, Zografou Campus, Athens, Greece; 3 Department of Marine Sciences, University of the Aegean, Mytilene, Greece; 4 Hellenic Agricultural Organization-DIMITRA, Fisheries Research Institute, Kavala, Greece; 5 Department of Ecology, School of Biology, Aristotle University of Thessaloniki, Thessaloniki, Greece; 6 Environmental Microbiology and Biotechnology Center, Agricultural Research Institute, Nicosia, Cyprus

**Keywords:** 16S rRNA gene, 18S rRNA gene, bacterioplankton, filtration, high-throughput sequencing, marine microbiota, passive sampling, Sterivex

## Abstract

**Introduction:**

Environmental DNA (eDNA) and high-throughput sequencing are now common tools for investigating marine microbial communities in coastal environments. Nevertheless, different eDNA sampling workflows are used across studies, while direct comparisons of their outputs under the same environmental and analytical framework remain limited.

**Methods:**

We aimed to compare three eDNA sampling approaches, two active filtration workflows using membrane filtration (Filter) and Sterivex cartridges (Sterivex), and one passive Gauze-based workflow, applied simultaneously in three coastal gulfs of Greece, to examine whether sampling workflow influences the recovered bacterial and eukaryotic diversity and community structure via 16S and 18S rRNA gene metabarcoding. The workflows differed in sampling characteristics, including pre-screening, processed water volume and capture mechanism, with 200-5m pre-screening applied only to Filter and Sterivex.

**Results:**

Filter and Sterivex produced concordant patterns of community structure and generally lower between-sample variability. They were nonetheless not fully equivalent: differences in diversity, taxonomic representation and network hub identity indicate that the two active workflows recover partially distinct community representations. Gauze produced a more variable and compositionally distinct signal, particularly for eukaryotic communities, where concordance with the active workflows was weak and occasional low-yield recovery occurred. Workflow-related differences were also observed in exploratory co-occurrence network topology and hub identity, indicating that workflow choice can influence downstream ecological interpretation.

**Discussion:**

Each workflow appeared to be suitable for different research objectives, with passive sampling providing complementary information. Maintaining a consistent sampling workflow is therefore particularly important for monitoring programs and cross-study comparisons.

## 1 Introduction

The combination of environmental DNA(eDNA) sampling and high-throughput sequencing (HTS) has shown rapid development over the last two decades, revolutionizing marine diversity investigations (Logares et al., 2014; Patin and Goodwin, 2023). The use of molecular markers, such as the 16S and 18S rRNA genes, has allowed the characterization of prokaryotic and eukaryotic communities across a wide range of marine ecosystems, contributing to the understanding of marine microbiota roles and functions (Sunagawa et al., 2015). Despite this advancement, the methodological heterogeneity of procedural choices during sampling, such as filtration strategy, filter pore size and material, processed water volume, and sample preservation can create bottlenecks for cross-study comparability (Patin and Goodwin, 2023). Although international initiatives are working toward standardizing marine -omics workflows, systematic evaluations of different eDNA sampling methods remain limited (Patin and Goodwin, 2023). As a result, researchers lack the evidence-based guidance needed to select the most fit-for-purpose approach to address specific ecological questions in variable environmental contexts.

While other capture methods exist, such as precipitation, centrifugation, or tangential flow capture, active filtration using membrane and pump-based systems is the standard approach for targeting marine microbial diversity, allowing for flexible volume processing and direct integration into various molecular sequencing workflows (Padilla et al., 2015). For this, aquatic microbial ecologists widely utilize flat and cartridge membrane filters Pascoal et al., (2023). Researchers commonly employ vacuum filtration to pull water through a disc, and peristaltic filtration to move water through tubing and into enclosed or flat filters (Yamahara et al., 2025). Open flat membrane filters allow the processing of defined water volumes, and the use of selected filter pore sizes. When applied sequentially, they can also enable size fractionation of microbial communities across different particle-size classes (Padilla et al., 2015; Torres-Beltrán et al., 2019). This method has led to the characterization of rare and abundant microbial eukaryotes in different plankton size fractions (Logares et al., 2014) and has assisted global ocean surveys such as Tara Oceans, where large microbial gene catalogs have been constructed and broad biogeographic patterns in ocean microbiomes are identified (Sunagawa et al., 2015). Open filters have also been applied *in situ*, revealing active microbial groups involved in processes such as sulfur cycling and carbon fixation (Torres-Beltrán et al., 2019). Additionally, filtration-based approaches have been used to examine anthropogenic source effects on marine microbial diversity (Skylaki et al., 2026) and assess pollutant impacts on complex planktonic communities using network analysis (Genitsaris et al., 2023).

Sterivex filtration is a cartridge-based form of active filtration that uses enclosed filters instead of open membranes, providing ease of use in the field, reduced direct handling and environmental exposure of the filter, and compatibility with on-site fixation (Wong et al., 2020; Anderson and Thompson, 2022). Sterivex-based sampling has supported studies of microbial responses to environmental gradients and human impacts in marine systems. For example, it has been used in a Swedish fjord system targeting bacterial and fungal communities under micropollutant pressures (Acosta et al., 2026). In remote oligotrophic regions like the North Pacific Subtropical Front, Sterivex-based sampling has been used to examine horizontal distribution of microbes and their relationships with temperature, depth, and longitude, while also supporting insights into microbial symbiosis and evolution (Lopes et al., 2024). Cartridge filtration has contributed to multi-taxonomic assessments, showing gradual shifts in fish and eukaryotic communities and sharp bacterial transitions along salinity thresholds (Hamamoto et al., 2025).

Passive collection workflows have received increasing attention in recent years since they require little equipment and can be deployed under simple field conditions. In contrast to active filtration, passive collection does not process a defined water volume but accumulates suspended biological material by submerging a device containing sorptive materials, including gauze, artificial cellulose sponges, or polymer membranes, directly into the water column for a defined exposure period (Karamati et al., 2024). The use of passive sampling in marine microbial applications remains limited, with most available work focusing on pathogen or bacterial detection, or broader macro-biodiversity surveys rather than whole marine microbial community assessments (Karamati et al., 2024; Kressler et al., 2026). A small number of comparison studies highlight that passive sampling provides a microbial community signal which is not interchangeable to that obtained through active filtration workflows (Saltonstall et al., 2024; Qian et al., 2026).

Existing methodological comparisons provide useful information on individual components of marine microbial eDNA sampling, including filtration volume (Padilla et al., 2015; Torres-Beltrán et al., 2019), filter type (Pascoal et al., 2023), pore-size fractionation (Pascoal et al., 2023; Gregori et al., 2025), and extraction workflow (Anderson and Thompson, 2022; Gregori et al., 2025). However, direct comparisons of active membrane filtration, Sterivex filtration, and passive gauze collection applied simultaneously to the same marine microbial samples are not yet available. This is particularly relevant when bacterial and microbial eukaryotic communities are analyzed together under the same environmental conditions. As a result, there is still limited evidence to guide method selection or to determine whether methodological choice alters ecological interpretation. Thus, we aimed to compare three eDNA sampling workflows, active membrane filtration using an electric pump, Sterivex closed-system filtration, and passive gauze-based collection, applied to seawater samples collected from three coastal gulfs of Greece. These workflows include inherent differences in pre-screening, processed water volume, particle-size fractions and capture material. The sampled areas encompass a broad gradient of anthropogenic stressors, including varying combinations of urban, industrial, maritime, and agricultural pressures, setting the ground for testing the selected eDNA sampling strategies across a broad range of marine microbiota variability. Using 16S and 18S rRNA metabarcoding, we (1) examined whether the three workflows yield different diversity and community composition estimates for bacterial and eukaryotic communities, (2) explored within-workflow variability and the community structure concordance, and (3) evaluated how workflow choice reflects microbe-microbe interactions assessed through network associations.

## 2 Materials and methods

### 2.1 Study sites and sampling

The Saronikos Gulf, adjacent to the metropolitan area of Athens, receives strong anthropogenic inputs from dense urbanization, intense maritime traffic, port activities, and the Psyttalia Wastewater Treatment Plant (WWTP). Additionally, industrial activities are concentrated mainly around Elefsis Bay, including refineries, shipyards and chemical, or metal-related industries (Pavlidou et al., 2019; Kalloniati et al., 2023; Skylaki et al., 2026). The Thermaikos Gulf, bordering the metropolitan area of Thessaloniki, is impacted by urban and industrial wastewater, port operations, agricultural and livestock runoff, mussel farming, and intensive fisheries. These are combined with substantial nutrient loading from transboundary riverine inflows, contributing to chronic enrichment and causing frequent harmful algal blooms (Genitsaris et al., 2019; Kouvara et al., 2025). The Kavala Gulf, located in Kavala, one of the biggest cities in northeastern Greece, represents a smaller but locally impacted coastal system, affected by urban and tourism-related activities, fishing and aquaculture, ship-related operations, and industry linked mainly to oil-related facilities and phosphate fertilizer production, including associated waste deposits and metal contamination (Noli et al., 2024; Tsamos et al., 2025).

Water sampling was carried out during a dedicated cruise for each gulf. Saronikos Gulf was sampled on 22 April 2024, Thermaikos Gulf on 16 June 2024, and Kavala Gulf on 18 June 2024. A total of 108 samples were collected. In each gulf, six sampling sites were selected (Figure 1; Supplementary Table S1) to capture spatial variability. Samples were collected from the surface and the lower boundary of the euphotic zone in each site. Euphotic depth (*Z*_*eu*_) was estimated from Secchi disk visibility (*Z*_*SD*_) as *Z*_*eu*_ = *f* × *Z*_*SD*_, with *f* = 2.5, following the empirical relationship commonly used to approximate the 1% light depth in marine waters (Strickland, 1958). For each sampling, three workflows were employed to assess potential differences in capturing marine microbial diversity and community structure patterns: (i) active filtering with a vacuum pump (Filter), (ii) syringe sterivex filtering (Sterivex), and (iii) gauze passive sampling (Gauze). For the active filtrations, seawater was first collected with a Niskin bottle and, in accordance with standard filter-based sampling protocols, sieved through a 200 μm mesh to exclude large particles and metazoans (Ruiz-González et al., 2019; Sánchez et al., 2024). The water was then filtered through 0.22 μm pore-size polycarbonate Nuclepore membrane filters (Grade 482) or 0.22 μm pore-size, PVDF Sterivex cartridges (Merck Millipore), respectively, until clogged. For the passive filtration, a gauze sheet was mounted on a 3D-printed sampler (Maiello et al., 2022) without a pre-screening step and immersed at the sampling site for 10 min, allowing seawater to flow through. The choice for a 10-min deployment of the passive sampler is an arbitrary trade-off targeting to capture the bulk of microbial diversity without compromising the spatial restrictions of planktonic microbial populations. All filters and gauze samples were immediately stored at −20 °C until DNA extraction.

**Figure 1:**
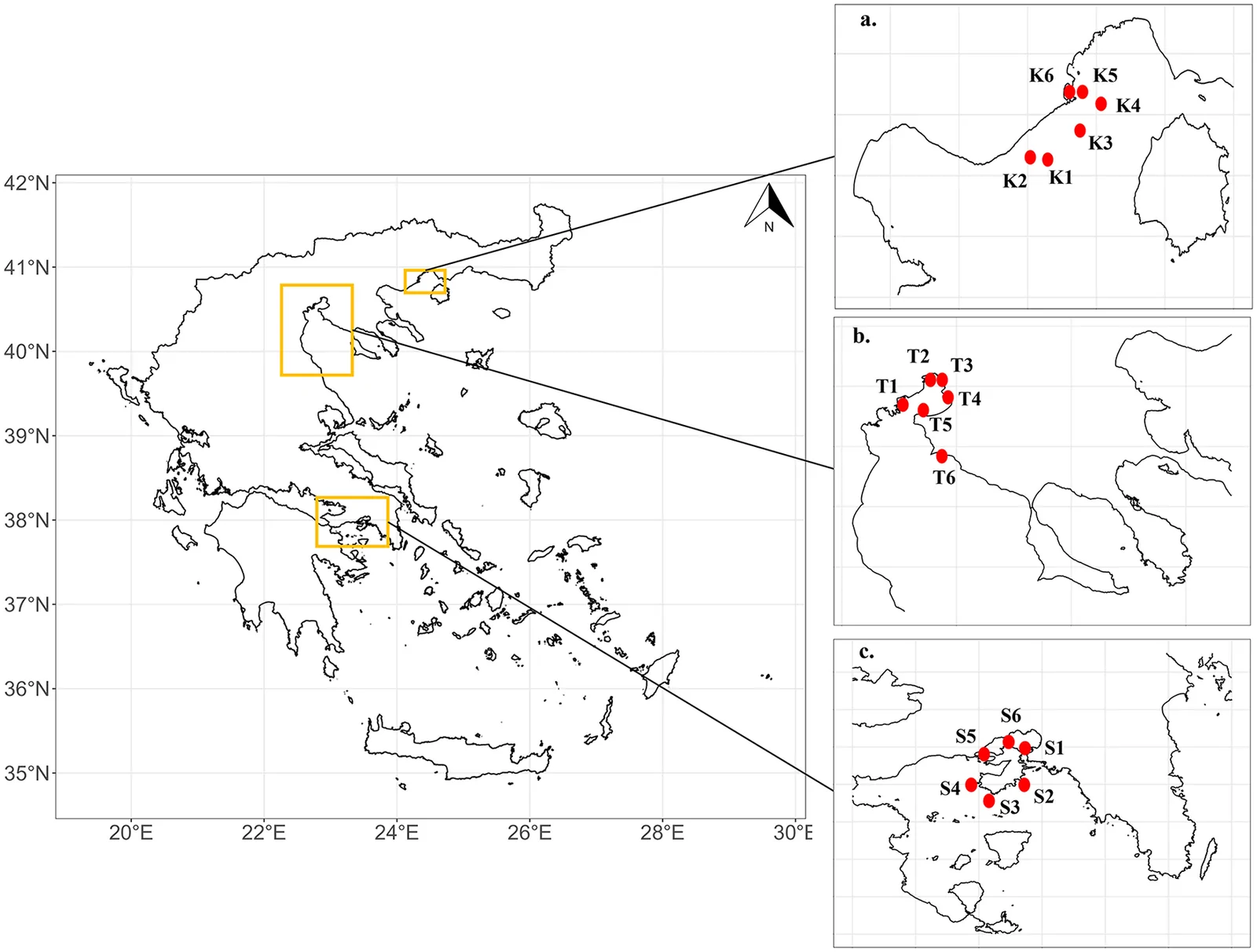
Locations of the sampling sites in **(a)** Kavala, **(b)** Thermaikos and **(c)**. Saronikos Gulfs.

### 2.2 High throughput sequencing and bioinformatic pipelines

The eDNA was extracted following standard protocols using mechanical lysis (Genitsaris et al., 2023; Gubili et al., 2025). Sterivex cartridges were first opened to remove the enclosed membrane. Open membrane filters, gauze material, and the removed Sterivex membranes were then transferred into bead-beating tubes containing lysis buffer. Then, samples were subjected to combined mechanical and chemical lysis, purification of the lysate and removal of contaminants and inhibitors, DNA binding to a silica membrane, washing, and final elution of the purified DNA. A blank extraction control was processed separately prior to sequencing. DNA concentration and quality were assessed using a ThermoScientific™ NanoDrop™ (2000) spectrophotometer (Thermo Scientific, Waltham, MA, USA) together with Qubit4 fluorometer with a Qubit dsDNA HS Assay kit (Invitrogen, ThermoFisher). PCR amplification targeted two marker regions. The V3–V4 hypervariable region of the 16S rRNA gene for bacterioplankton, using primers S-DBact-0341-b-S-17 (CCTACGGGNGGCWGCAG) and S-D-Bact-0785-a-A-21 (GACTACHVGGGTATCTAATCC) (Klindworth et al., 2013), and the V4 region of the 18S rRNA gene for planktonic protists, using primers E572F (CYGCGGTAATTCCAGCTC) and E1009R (AYGGTATCTRATCRTCTTYG) (Comeau et al., 2011).

Amplicons were sequenced on an Illumina MiSeq platform using 2 × 300 bp paired-end chemistry, which provides overlapping reads that can be merged into full-length sequences. PCR amplification and sequencing were carried out at the Mr. DNA Molecular Research Laboratory (Shallowater, USA) following in-house standard protocols. A mock-community positive control and a no-template PCR control (NTC) were included during amplification, together with technical PCR duplicates for quality control. The NTC yielded no sequencing reads.

Raw amplicon reads were processed using mothur v1.48.3 following the MiSeq standard operating procedure (Schloss et al., 2009, 2011). Forward and reverse reads were merged, and contigs containing ambiguous nucleotide calls (maxambig = 0), exceeding 450 bp in length, or containing >8 bp homopolymers were discarded. Sequences were dereplicated into unique reads and aligned independently against the SILVA SSU Ref NR99 database, release 138.1 (Quast et al., 2013). Putative chimeric sequences were identified and removed using the UCHIME algorithm (Edgar et al., 2011). Non-chimeric reads were clustered into Operational Taxonomic Units (OTUs) at 97% sequence similarity using the average neighbor method. OTUs represented by a single read across the entire dataset were removed to minimize artifactual diversity. Taxonomic assignment was performed using SINA searches against the SILVA database for bacteria (Pruesse et al., 2012) and the classify.seqs command in mothur against the Protist Ribosomal Reference database (PR2, v5.0.0; Guillou et al., 2013) for eukaryotes, applying a minimum similarity threshold of 80% to the closest reference. In the eukaryotic dataset, one Gauze sample (G_T5s) was excluded during mothur processing, while two more (G_K4s and G_K4d) did not provide sufficient data for complete alpha-diversity calculation and were therefore excluded from alpha-diversity analyses. Amplicon raw reads were submitted to the NCBI Sequence Read Archive (SRA) under BioProject accession PRJNA1496876.

### 2.3 Statistical analysis

#### 2.3.1 Community structure

Rarefaction curves were generated separately for bacterial and eukaryotic datasets in *R* (v4.4.1) (R Core Team, 2024) using the rarecurve function of the vegan (v2.7.3; Oksanen et al., 2026) package, with a step size of 100 reads. Alpha-diversity indices, including OTU richness (S), Shannon (H′), Simpson (1—D) and Pielou's Evenness (J′) were computed using the phyloseq package (v 1.48.0) (McMurdie and Holmes, 2013) in R. D = Σp_*i*_^2^ denotes Simpson's dominance index based on proportional species abundances, while Pielou's index was calculated as the ratio of H′ to the natural logarithm of the OTU richness (ln(S)). Calculations were based on untransformed read counts. We chose not to perform a subsampling step to a common depth, as sequencing yield was considered part of the overall performance of each sampling workflow. Beta-diversity was assessed using Bray–Curtis dissimilarities calculated from relative abundance data within the phyloseq package. To examine community structure independently for each sampling workflow, non-metric multidimensional scaling (NMDS) ordinations were generated separately for Filter, Sterivex and Gauze for each microbial fraction, using two dimensions (*k* = 2) and a maximum of 100 random starts.

#### 2.3.2 Statistical comparison of sampling workflows

Alpha-diversity indices were compared among sampling workflows using distribution-specific generalized linear mixed-effects models fitted with the glmmTMB package (v 1.1.14) in *R* (Brooks et al., 2017). Method variable, representing the 3 workflows, was included as a fixed effect, while station × depth × location was included as a random intercept to account for the paired sampling design (response ~ Method + (1 | Block)). No interaction terms were included. Observed OTU richness was modeled using a negative-binomial distribution, Shannon using Gamma distributions with log links, and Simpson and Pielou's evenness using beta distributions with logit links. Bacterial evenness values were adjusted to fall within the open interval (0, 1) before beta regression. For bacterial models, dispersion was additionally allowed to vary among methods. The overall effect of Method was assessed using type-II Wald χ^2^ tests for bacterial models and using likelihood-ratio tests for eukaryotic models, with Benjamini–Hochberg correction (Benjamini and Hochberg, 1995) across indices. *Post-hoc* pairwise contrasts among methods were calculated using estimated marginal means with Benjamini–Hochberg correction within each index (Lenth and Piaskowski, 2026). Model-based pairwise effect estimates with 95% confidence intervals are provided in Supplementary Table S2. Workflow-related differences in variability were assessed separately using median-centered Levene tests on residuals from Gaussian linear mixed-effects models with the same fixed- and random-effects structure (Bates et al., 2015), while relative variability was summarized descriptively using the coefficient of variation (CV% = 100 × SD/mean) for each workflow and index. To assess whether the different sampling workflows recovered concordant patterns of community structure across the same environmental sampling units, pairwise Procrustes analyses were performed between the workflow-specific NMDS configurations (Gower, 1975). Corresponding samples were matched across workflows, and symmetric Procrustes rotation was applied for the Filter–Sterivex, Filter–Gauze and Sterivex–Gauze comparisons. The strength of concordance between each pair of ordination configurations was quantified using the Procrustes correlation, and its statistical significance was evaluated using PROTEST with 9,999 permutations (Peres-Neto and Jackson, 2001; Oksanen et al., 2026). Inspection of post-processing library sizes additionally identified one bacterial sample (G_T3s) and one eukaryotic sample (G_K1s) as effectively empty for community-composition analyses. These samples were excluded from beta-diversity analyses.

#### 2.3.3 Network analyses

Individual co-occurrence networks were constructed for the OTUs captured by each sampling workflow across the studied gulfs, using 12 samples per workflow-by-gulf combination (except for the Thermaikos Gauze eukaryotic network, with 11 samples). To ensure a focus on the core community, OTUs representing > 0.1% mean relative abundance within each specific method-location sub-dataset were retained (Kourkoutmani et al., 2025). Pairwise statistical dependencies were characterized using the maximal information-based non-parametric exploration (MINE) statistic, with the maximal information coefficient (MIC) providing a score that represents the strength of the relationship independently of the association type (e.g., linear or exponential) (Reshef et al., 2011). Permutation-derived *p*-values were adjusted for multiple testing using the Benjamini–Hochberg false discovery rate (FDR) procedure. Associations with adjusted *p* < 0.05 and MIC > 0.8 were retained as network edges (Reshef et al., 2011; Genitsaris et al., 2016). The tested OTU pairs and retained associations for each network are provided in Supplementary Table S3. Topological parameters were calculated for each network to describe its overall structure. Among them, the Number of Nodes described network size, whereas Network Density and Average Number of Neighbors indicated overall connectivity. Network Diameter and Average Shortest Path Length reflected network compactness, while Centralization and Heterogeneity described the extent to which links were concentrated around a few highly connected OTUs. The Clustering Coefficient indicated the tendency of OTUs to form local groups. The 10 nodes with the highest number of network connections were identified as putative hubs (Lee et al., 2022). Statistical computations, network construction, and topological assessments were performed, using the packages minerva (v 1.5.10) and igraph (v 2.2.2) (Albanese et al., 2013; Csárdi et al., 2026).

## 3 Results

### 3.1 Microbial diversity and community composition

Rarefaction curves for bacterial communities generally plateaued across sampling methods (Supplementary Figure S1). On the other hand, eukaryotic curves were less consistently saturated, particularly for Gauze, with some samples retaining an increasing trajectory with the sequencing coverage achieved (Supplementary Figure S2). Given that alpha-diversity indices were calculated without subsampling to a common sequencing depth, observed richness is interpreted here as workflow-specific recovered richness.

#### 3.1.1 Bacterial alpha-diversity

Alpha-diversity estimates exhibited high variability among sampling workflows (Figure 2; Supplementary Table S4). Filter samples contained 159–351 OTUs and generally yielded lower Shannon diversity (2.58–3.93), Simpson diversity (0.81–0.96), and Pielou's evenness than the other methods. Gauze was characterized by the greatest variability across all indices and included both the lowest and highest values observed in the dataset, ranging from 2 to 1,832 OTUs and from 0.69 to 5.17 in Shannon diversity. This broader spread was evident across all three gulfs but was particularly pronounced in Saronikos and Thermaikos. In contrast, Sterivex samples captured a narrower diversity range, with observed OTU richness ranging from 101 to 361, Shannon values from 3.12 to 4.53, and Simpson values from 0.83 to 0.98.

**Figure 2:**
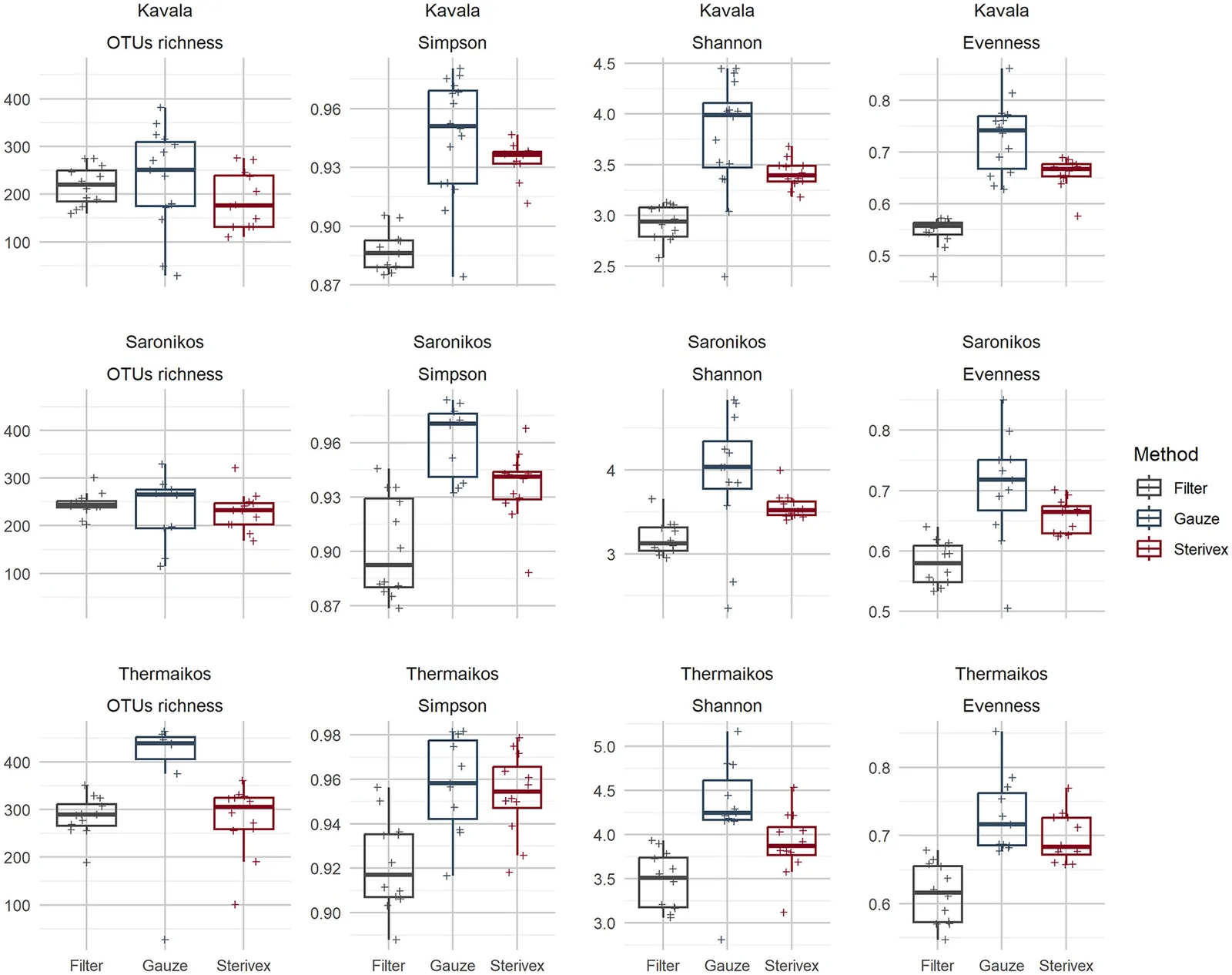
Distribution of the alpha-diversity indices of bacterial communities in the examined locations. The horizontal line depicts the median values, and the dots depict individual samples. Outliers were filtered per-index using 1.5xIQR thresholds for visualization purposes.

#### 3.1.2 Bacterial relative abundance

Across all workflows and locations, bacterial communities were mainly composed of Alphaproteobacteria, Gammaproteobacteria, Cyanobacteria, and Bacteroidia, although their relative contributions varied among sampling workflows within each gulf (Figure 3). Across gulfs, Filter and Sterivex showed broadly similar dominance structures, but Sterivex generally displayed reduced Alphaproteobacteria contributions and increased Gammaproteobacteria and/or Bacteroidia compared with Filter, as illustrated in Kavala gulf (Alphaproteobacteria: 53.0% to 43.8%; Gammaproteobacteria: 10.2% to 21.8%) and Saronikos (Alphaproteobacteria: 46.7% to 28.7%; Bacteroidia: 14.5% to 23.8%). In contrast, Gauze diverged from the other two methods in the dominant class hierarchy. Gammaproteobacteria exceeded Alphaproteobacteria in Kavala Gauze samples (28.6% vs. 24.0%), while Bacteroidia and Gammaproteobacteria both exceeded Alphaproteobacteria in Thermaikos (23.5% and 18.4% vs. 17.1%). Additional classes, including Actinobacteria, Bacilli, and Phycisphaerae in Kavala, Kiritimatiellae and Bacilli in Saronikos, and Bdellovibrionia, and Desulfuromonadia in Thermaikos were represented above the 2% visualization threshold in Gauze samples, while remained below this threshold or were not detected in the other workflows.

**Figure 3:**
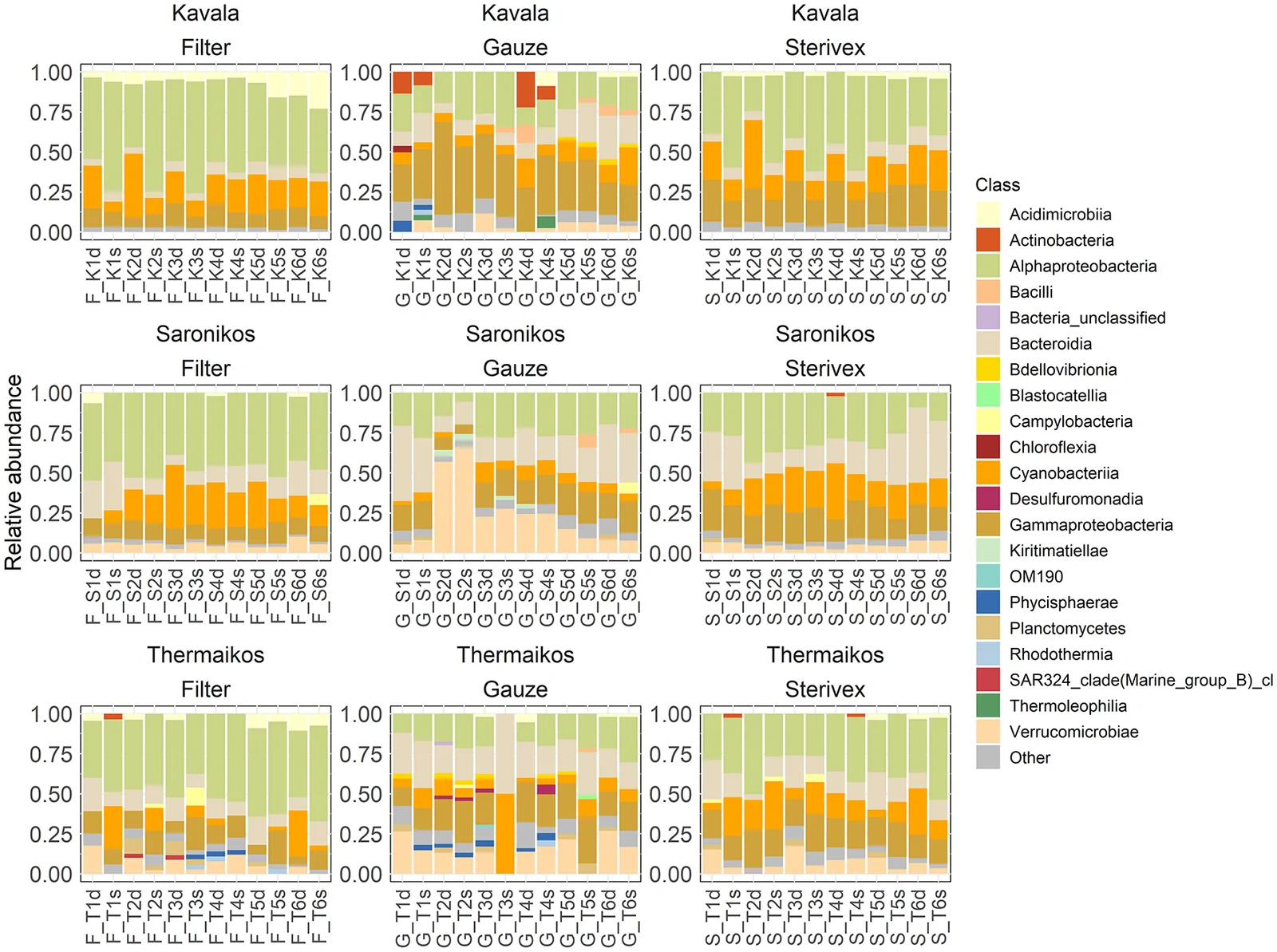
Relative abundance of bacterial classes and unclassified bacterial reads across sampling workflows and locations. Classes contributing each < 2% are grouped as “Other.” See Supplementary Table S1 for sampling code annotations.

#### 3.1.3 Eukaryotic alpha-diversity

Eukaryotic alpha-diversity varied among sampling workflows, although method-related patterns were less consistent across gulfs than in the bacterial dataset (Figure 4; Supplementary Table S4). Filter recovered a substantial number of OTUs (97–270 OTUs), partly overlapping with Sterivex in richness values. However, Shannon (0.58–3.16), Simpson (0.17–0.90), and Pielou's evenness (0.12–0.59) were frequently lower than with Sterivex. Sterivex often captured the highest, or among the highest, alpha-diversity values, with observed richness ranging from 19 to 494 OTUs, Shannon from 1.13 to 4.18 and Simpson's index from 0.40 to 0.95. The highest values were observed in Kavala gulf (max 494 OTUs; Shannon 4.18) and several Thermaikos samples (max 416 OTUs; Shannon 3.98). Gauze exhibited the strongest gulf-dependent variation and was the only method with three unresolvable samples because of insufficient read recovery after quality filtering (Supplementary Table S4). Gauze diversity remained relatively low in Kavala gulf (4–63 OTUs; Shannon 0.60–2.48), spanned the widest variation and the lowest evenness values in Saronikos gulf (8–148 OTUs; Shannon 0.47–3.17), and increased in Thermaikos gulf (54–287 OTUs; Shannon 2.08–3.46), where values approached those obtained with Sterivex.

**Figure 4:**
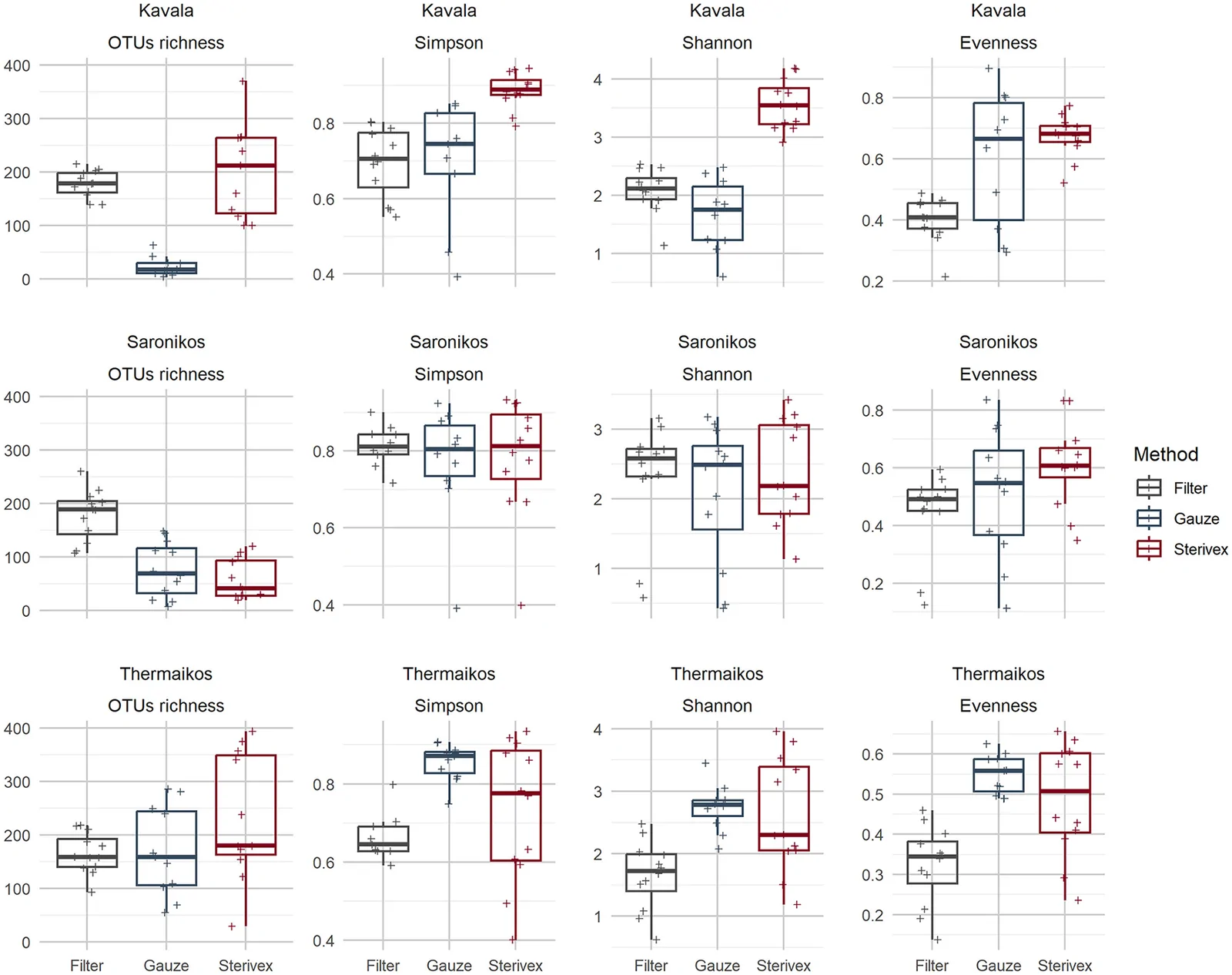
Distribution of the alpha-diversity indices of eukaryotic communities in the examined locations. The horizontal line depicts the median values, and the dots depict individual samples. Outliers were filtered per-index using 1.5xIQR thresholds.

#### 3.1.4 Eukaryotic relative abundance

In eukaryotic communities, Filter and Sterivex retained more similar taxonomic profiles within each gulf, with Dinophyceae representing the dominant group in both methods (Filter: 40.5–67.3%; Sterivex: 31.3–55.8%) (Figure 5). The main difference was the consistent enrichment of Syndiniales in Sterivex compared with Filter across all gulfs (Sterivex: 10.2–21.8%; Filter: 5.1–7.4%). A secondary difference was the broader protist signal detected by Sterivex, including Sagenista, Chrysophyceae and Chlorodendrophyceae, which were lower or absent in Filter samples. Conversely, Mamiellophyceae and Cryptophyceae were detected only in Filter, albeit only in Saronikos gulf. Gauze differed from both active filtration workflows and showed greater within-gulf compositional variability, capturing lower proportions of Dinophyceae (16.4–34.4%) and higher contributions of metazoan classes, most notably Cnidaria (17.8–41.9%) and Arthropoda, the latter being particularly elevated in the gulf of Thermaikos (17.5%). Spirotrichea also appeared recurrently across Gauze samples from all gulfs.

**Figure 5:**
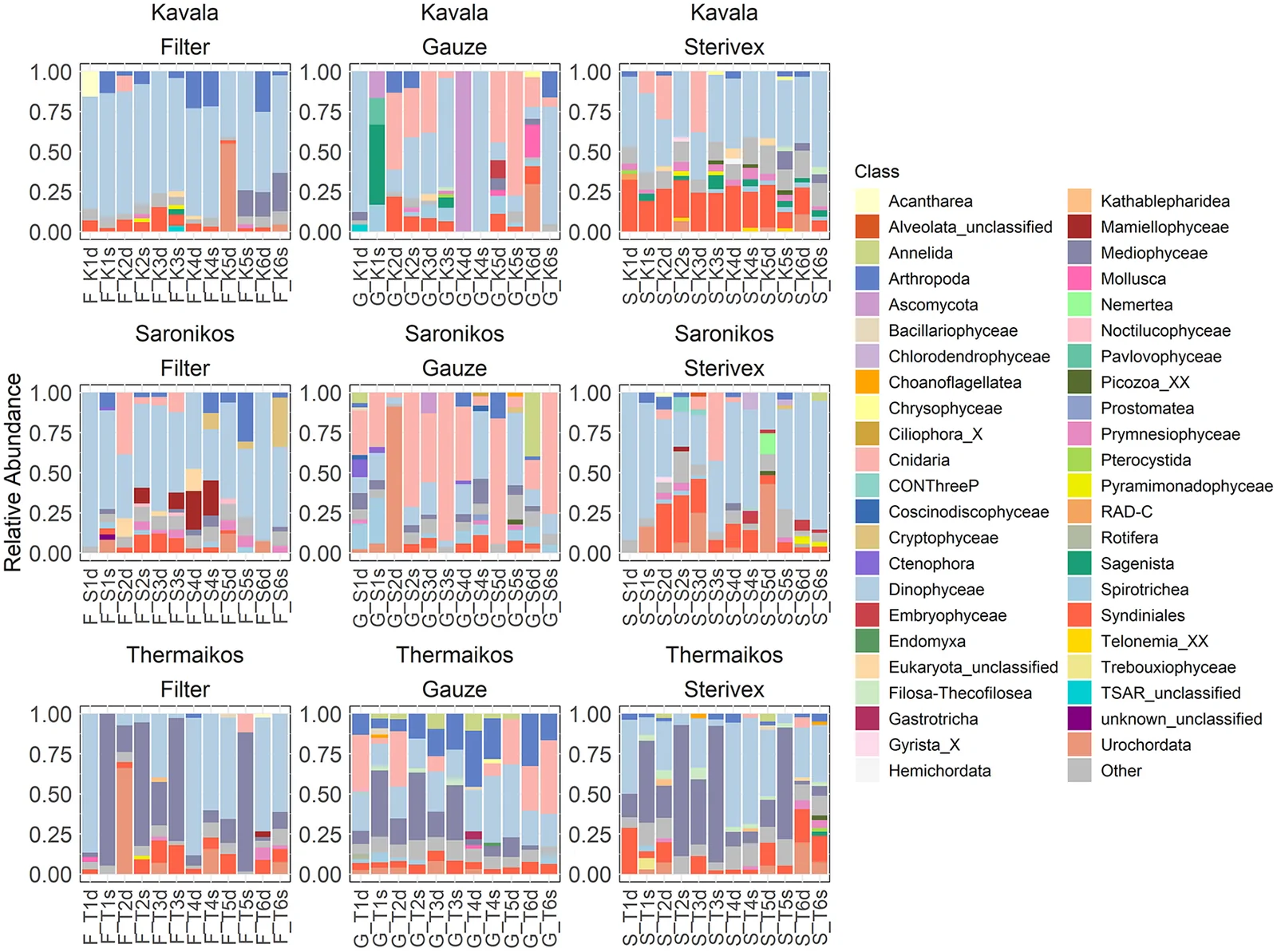
Relative abundance of eukaryotic classes and unclassified eukaryotic reads across sampling workflows and locations. Classes contributing each < 2% are grouped as “Other.” See Supplementary Table S1 for sampling code annotations.

### 3.2 Statistical comparison of sampling workflows

Regarding bacterial communities, the distribution-specific generalized linear mixed-effects models indicated that the three workflows do not produce equivalent alpha-diversity estimates across all indices (all *p* < 0.01; Table 1; Supplementary Table S2). Based on pairwise contrasts, Gauze was generally associated with higher diversity estimates than the other methods, whereas Filter and Sterivex were often closer in values for richness. Given the inherent dependence of Pielou's evenness to richness, the higher evenness estimates associated with the passive workflow partly reflect the very low richness of some Gauze samples, rather than consistently more even bacterial communities. The stability analysis revealed that the workflows also differed in variability. Residual dispersion differed significantly among methods for OTU richness, Shannon, and Evenness (median-centered Levene tests on Gaussian mixed-model residuals; all *p* ≤ 0.006), but not for Simpson (*p* = 0.116). Gauze was substantially more variable, especially for richness, whereas Filter and Sterivex were more consistent, showing lower dispersion (Table 1; CV). Between the latter two workflows, Filter showed lower CV values for richness, while Sterivex showed the lowest CV values for Shannon, Simpson and Evenness. Procrustes analyses showed significant concordance among all three workflow-specific NMDS configurations (Figure 6). The strongest correspondence was observed between Filter and Sterivex (Procrustes correlation *r* = 0.821, m12^2^ = 0.325, *p* < 0.001), followed by Filter and Gauze (*r* = 0.641, m12^2^ = 0.589, *p* < 0.001) and Sterivex and Gauze (*r* = 0.601, m12^2^ = 0.638, *p* < 0.001).

**Table 1:** Alpha-diversity workflow summary.

Bacteria						
Alpha index	BH-adjusted *p*	Pattern	Variance_differs	Levene_*p*	Lowest-CV%	CV
OTU richness	0.009^**^	Gauze^a^, Filter^b^, Sterivex^b^	Yes	< 0.001^***^	Filter	18.4
Shannon	< 0.001^***^	Gauze^a^, Sterivex^a^, Filter^b^	Yes	0.006^**^	Sterivex	8.6
Simpson	< 0.001^***^	Sterivex^a^, Gauze^ab^, Filter^b^	No	0.116	Sterivex	2.8
Evenness	< 0.001^***^	Gauze^a^, Sterivex^b^, Filter^c^	Yes	< 0.001^***^	Sterivex	5.3
**Eukaryotes**						
OTU richness	< 0.001^***^	Filter^a^, Sterivex^a^, Gauze^b^	Yes	< 0.001^***^	Filter	21.5
Shannon	< 0.001^***^	Sterivex^a^, Gauze^b^, Filter^b^	No	0.201	Sterivex	30.8
Simpson	0.002^**^	Sterivex^a^, Gauze^ab^, Filter^b^	No	0.620	Sterivex	18.7
Evenness	< 0.001^***^	Sterivex^a^, Gauze^a^, Filter^b^	No	0.094	Sterivex	24.4

Pattern shows significant pairwise differences, with Methods ordered from higher to lower alpha-diversity estimates; different superscript letters indicate significant differences, whereas shared letters indicate non-significant differences. Variance differs and Levene *p* indicate whether residual variability differed significantly among workflows and the corresponding median-centered Levene test *p*-value. Lowest-CV Method identifies the workflow with the lowest coefficient of variation (CV = 100 × SD/mean); lower CV indicates greater relative consistency. Filter = open membrane filtration; Sterivex = cartridge filtration; Gauze = passive gauze sampling. Significance codes: ^**^p < 0.01, ^***^p < 0.001. BH-adjusted *p* gives the Benjamini–Hochberg-adjusted *p*-value for the overall Method effect from the distribution-specific generalized linear mixed-effects models (type-II Wald χ^2^ tests for bacteria and likelihood-ratio tests for eukaryotes). Superscript letters ^a^ and ^b^ indicate statistical groupings based on post-hoc pairwise comparisons; workflows with different letters differ significantly (BH-adjusted *p* < 0.05), whereas workflows sharing the same letter do not differ significantly.

**Figure 6:**
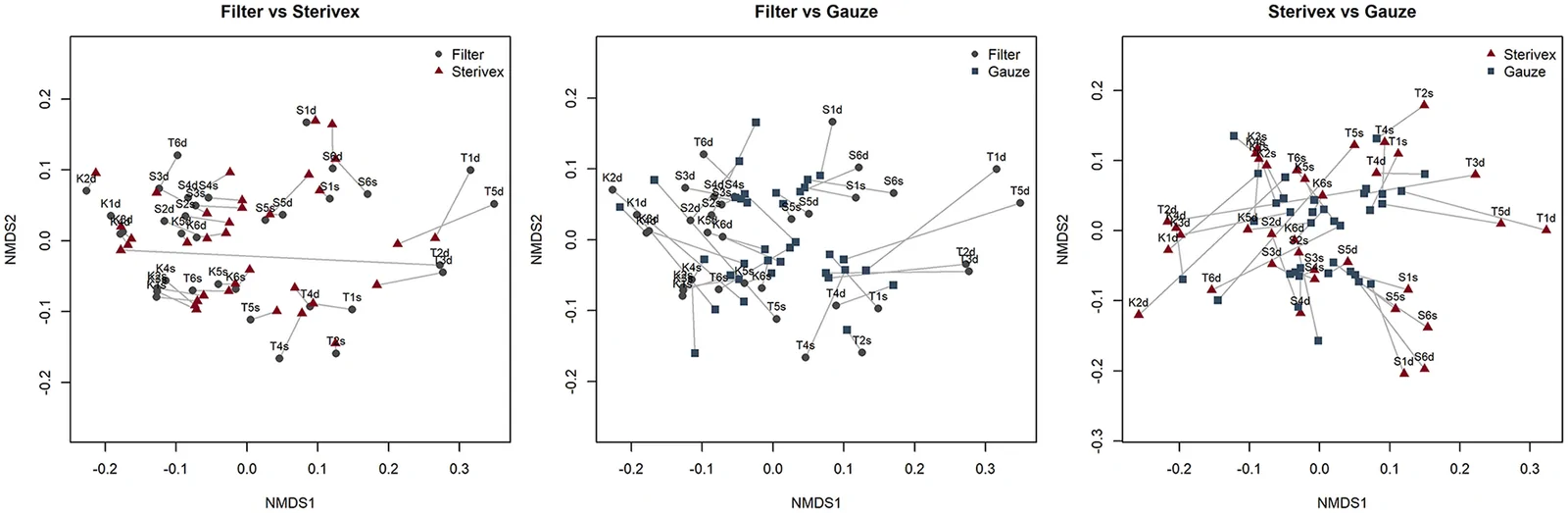
Pairwise Procrustes comparisons of bacterial community Non-metric Multidimensional Scaling (NMDS) configurations obtained with the three sampling workflows. Points represent corresponding sampling units recovered by each workflow, and connecting segments link matched samples between the two configurations. Filter, Sterivex and Gauze are indicated by different colors and symbols.

In eukaryotic communities, sampling workflows also systematically affected alpha-diversity (Table 1; Supplementary Table S2), with pairwise analyses highlighting different trends. For OTU richness, Gauze captured the lowest diversity, while Sterivex and Filter yielded similar values. For Shannon, Simpson and Evenness, Sterivex was the method capturing the highest values, with Gauze following and/or showing no statistical difference from Sterivex depending on the index. The Levene test indicated significant differences in residual dispersion among workflows for OTU richness (*p* < 0.001), but not for Shannon, Simpson or Evenness. Gauze exhibited the greatest variability for richness, whereas Filter was the most consistent for richness, and Sterivex showed the lowest CV for Shannon, Simpson, and Evenness. The comparison between Filter and Sterivex revealed the strongest Procrustes concordance (*r* = 0.655, m12^2^ = 0.571, *p* < 0.001). The concordance between Filter and Gauze was lower and not statistically significant (*r* = 0.318, m12^2^ = 0.899, *p* = 0.075), whereas the Sterivex–Gauze comparison resulted in a lower but statistically significant concordance (*r* = 0.355, m12^2^ = 0.874, *p* = 0.032) (Figure 7).

**Figure 7:**
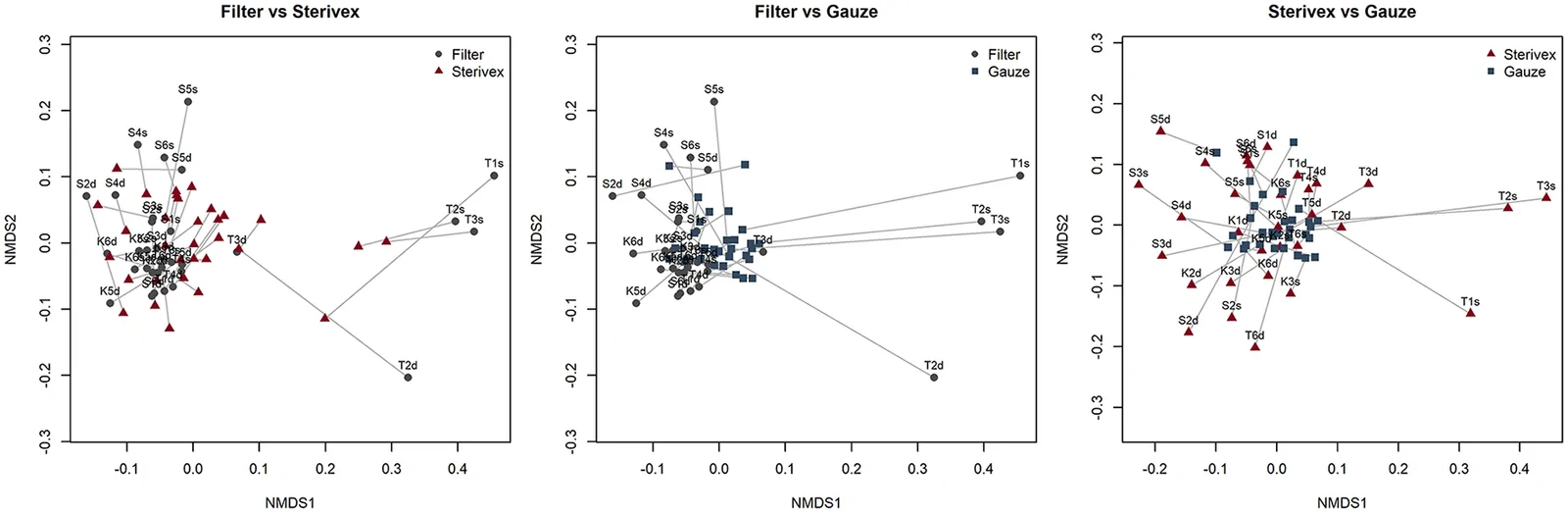
Pairwise Procrustes comparisons of eukaryotic community Non-metric Multidimensional Scaling (NMDS) configurations obtained with the three sampling workflows. Points represent corresponding sampling units recovered by each workflow, and connecting segments link matched samples between the two configurations. Filter, Sterivex and Gauze are indicated by different colors and symbols.

### 3.3 Network analyses

For bacterial communities, network topology varied among sampling workflows and gulfs without a consistent method-specific pattern across all locations (Table 2). In Kavala, Gauze contained the greatest Number of Nodes (112) and the highest Average Number of Neighbors (33.04), whereas Filter had the highest Network Density (0.34 vs. 0.30 for Gauze). In Saronikos, Gauze produced the largest and most connected network with 116 nodes, a Network Density of 0.45 and 51.97 Average Number of Neighbors. In Thermaikos, the strongest connectivity signal was represented by Sterivex, showing the highest values for both Network density and Average Number of Neighbors. The Clustering Coefficient was highest for Filter in all three gulfs, while other topological properties, including Average Shortest Path Length and Network Heterogeneity, varied among workflows and locations. Taxonomically, OTU overlap among bacterial top hubs was limited, although Filter and Sterivex shared highly connected OTUs in all three gulfs (Figure 8; Supplementary Table S5). These included Clade III- and S25–593-related OTUs in Kavala, *Prochlorococcus*-assigned Otu0006 in Saronikos, and NS5 marine group in Thermaikos. Alphaproteobacteria were recurrent among Filter and Sterivex hubs, particularly SAR116- and Rhodobacteraceae-related taxa. Gauze hub composition varied among gulfs and included several taxa not represented among the top hubs of either active workflow, including OM60(NOR5), OM43, OM190, AEGEAN-169, *Crocinitomix, Aurantivirga*, and *Roseibacillus*. Bacteroidia-related hubs were common to all workflows but were particularly prominent among Gauze hubs in Saronikos.

**Table 2:** Topological parameters characterizing the individual networks of each workflow per gulf.

	Bacteria								
	Kavala			Saronikos			Thermaikos		
	Filter	Gauze	Sterivex	Filter	Gauze	Sterivex	Filter	Gauze	Sterivex
No. of nodes	66	112	79	75	116	78	35	60	111
Network density	0.34	0.30	0.23	0.28	0.45	0.18	0.05	0.04	0.09
Avg. no. of neighbors	22.09	33.04	18.25	20.85	51.97	13.69	1.66	2.37	9.50
Network diameter	4	4	6	8	4	10	4	4	10
Network centralization	0.35	0.40	0.32	0.30	0.30	0.24	0.04	0.08	0.20
Shortest paths (avg)	1.78	1.82	2.16	2.40	1.64	2.86	1.37	1.42	3.50
Clustering coefficient	0.80	0.62	0.72	0.80	0.78	0.72	0.80	0.78	0.59
Network heterogeneity	0.57	0.51	0.60	0.74	0.44	0.76	0.48	0.68	0.72
	**Eukaryotes**								
	Filter	Gauze	Sterivex	Filter	Gauze	Sterivex	Filter	Gauze	Sterivex
No. of nodes	12	2	50	29	31	24	14	61	71
Network density	0.11	1.00	0.05	0.07	0.12	0.05	0.13	0.21	0.08
Avg. no. of neighbors	1.17	1.00	2.36	1.93	3.61	1.25	1.71	12.66	5.72
Network diameter	2	1	4	7	5	2	5	8	10
Network centralization	0.08	0.00	0.12	0.07	0.18	0.08	0.18	0.27	0.16
Shortest paths (avg)	1.22	1.00	1.78	2.67	2.71	1.21	2.23	2.42	3.58
Clustering coefficient	0.00	NA	0.56	0.39	0.47	0.43	0.40	0.68	0.62
Network heterogeneity	0.33	0.00	0.66	0.62	0.67	0.43	0.58	0.69	0.78

Avg., average; No., number; Filter, Active filtration with open filters; Sterivex, Active filtration with Sterivex cartridge; Gauze, passive filtration with gauze as sorptive material.

**Figure 8:**
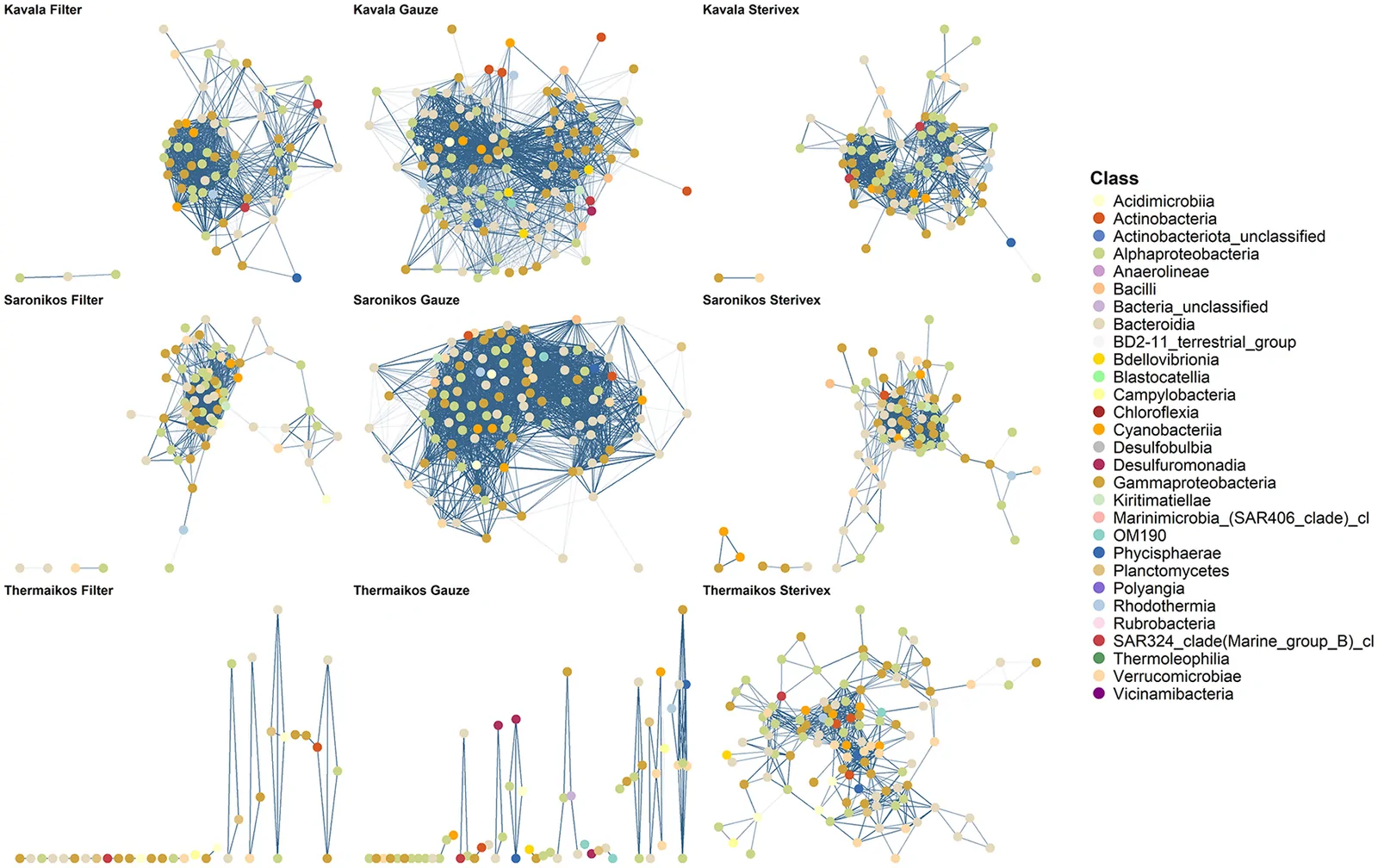
Network diagram of the bacterial Operational Taxonomic Units (OTUs; nodes) showing correlations (edges) with maximal information coefficient (MIC) values > 0.8 and Benjamini–Hochberg FDR-adjusted permutation *p* < 0.05. Different colors represent different Classes.

For eukaryotic communities, network architecture also varied among sampling workflows and gulfs (Table 2). In Kavala, Sterivex produced the largest network (50 Nodes) and the highest Average Number of Neighbors (2.36), whereas Filter had a higher Network Density (0.11 vs. 0.05). The Gauze network was reduced to only two nodes and was therefore not considered comparable with the other workflows. In Saronikos, Gauze produced the largest and most connected network, followed by Filter and Sterivex. In Thermaikos, Sterivex produced the largest network (71 Nodes), whereas Gauze showed the highest Network Density (0.21) and Average Number of Neighbors (12.66). Excluding the two-node Kavala network, Gauze showed the highest Centralization and Clustering Coefficients. At the taxonomic level, eukaryotic hub composition differed among sampling workflows within each gulf, with limited OTU overlap despite persistent representation of broader groups (Figure 9; Supplementary Table S5). In Kavala, Filter and Sterivex shared several hub classes, including Dinophyceae, Syndiniales, Mediophyceae, and Sagenista, but only one OTU (*Leptocylindrus*, Otu0063). Comparison with Gauze was restricted by its two-node network. In Saronikos, Dinophyceae, and Syndiniales represented top hubs with all three methods, but exact overlap remained limited between methods' pairs. Their remaining hub composition differed, with Gauze consisting of several diatom- and prymnesiophyte-related taxa, Filter including several dinoflagellates together with copepod-related OTUs, and Sterivex consisting of Acantharea, Chlorodendrophyceae and Cnidaria. In Thermaikos, all three workflows contained Dinophyceae and Syndiniales hubs, yet Filter and Sterivex shared no top-hub OTUs. Gauze shared one OTU with Filter and two with Sterivex, although the broader hub composition remained distinct, with Gauze including Cercozoa, Spirotrichea, Choanoflagellatea and green-algal groups, and Sterivex including Prymnesiophyceae, Picozoa, Pterocystida, and Sagenista.

**Figure 9:**
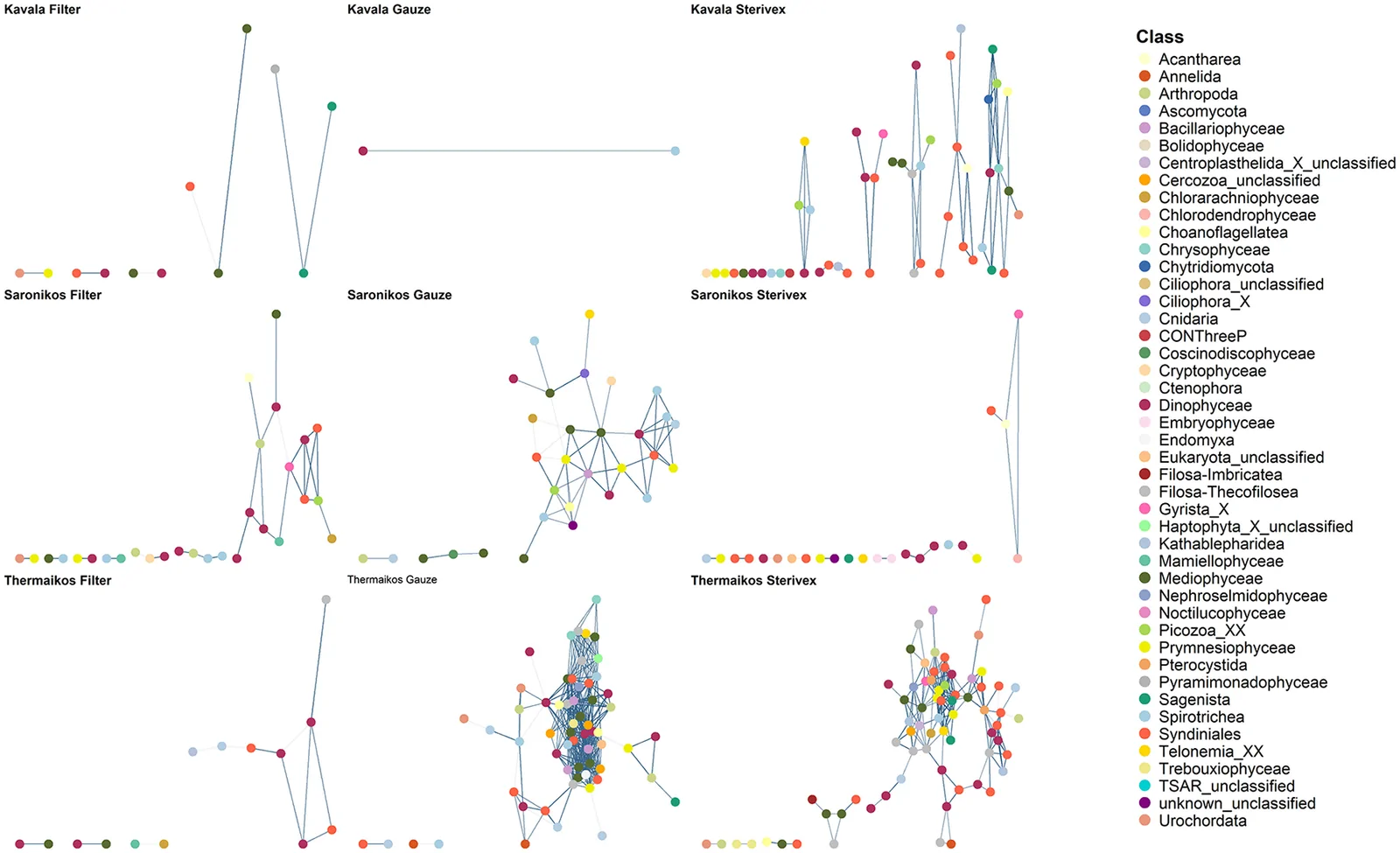
Network diagram of the eukaryotic Operational Taxonomic Units (OTUs; nodes) showing correlations (edges) with maximal information coefficient (MIC) values >0.8 and Benjamini–Hochberg FDR-adjusted permutation *p* < 0.05. Different colors represent different Classes.

## 4 Discussion

Although direct molecular studies of bacterioplankton remain limited for the studied gulfs, coastal marine bacterial communities are generally expected to be dominated by Alpha- and Gammaproteobacteria, together with Cyanobacteria and Bacteroidia, with relative proportions varying according to nutrient availability, riverine influence, urban inputs, aquaculture, and other anthropogenic pressures (Sunagawa et al., 2015; Šantić et al., 2021; Kalaitzidou et al., 2022). The bacterial profiles recovered here, mainly by the active workflows, were therefore consistent with the expected broad marine coastal signal. Similarly, the dominance of Dinophyceae in the eukaryotic communities, agrees with previous observations from productive Aegean coastal systems where dinoflagellates, diatoms and coccolithophores form recurrent components of the phytoplankton assemblage (Ignatiades, 2012; Androulidakis et al., 2021). Since each gulf was sampled on a different date, location-specific microbial communities are influenced by temporal succession.

Microbial diversity and community structure indicators, including alpha-diversity estimators, beta-diversity dissimilarities and network topological parameters, were influenced by sampling workflow choice. The observed differences represent the combined outcome of each workflow's individual steps, including pre-screening of larger particles and processed water volume rather than solely the isolated effect of each sampler. Passive Gauze sampling consistently produced different outputs from active Filter and Sterivex sampling across both bacterial and eukaryotic communities, combining high bacterial richness with greater between-sample variability and occasional eukaryotic low-yield failure. Unlike the active-filtration workflows, an intrinsic methodological constraint of the passive Gauze workflow was the lack of an equivalent 200 μm pre-sieving step. This difference may have contributed to the observed variance, particularly through greater retention of larger organism-derived material. Part of this variance may also reflect genuine ecological signal from a biological compartment that active filtration does not capture. Unlike active filtration, Gauze accumulates material through surface adsorption over deployment time rather than filtering a defined water volume, and may therefore integrate particle-associated microorganisms, extracellular DNA and surface-associated material (Bessey et al., 2022; Patin and Goodwin, 2023; Saltonstall et al., 2024). This mechanistic distinction was reflected taxonomically. In bacteria, Gauze samples reflected higher relative contributions of groups commonly associated with particle attachment, copiotrophy and organic-matter degradation, including Gammaproteobacteria and Bacteroidia-related lineages (Fernández-Gómez et al., 2013; Reintjes et al., 2023). In eukaryotes, the reduced dominance of Dinophyceae and the increased contribution of Cnidaria, Arthropoda, and Spirotrichea are consistent with possible retention of larger organisms, tissue particles, mucus and metazoan-associated material at the expense of the microbial fractions. This is reduced in filter-based methods due to the meshing step. Passive collectors may recover high microbial diversity but only partially overlap with active filtration datasets, supporting their interpretation as complementary rather than interchangeable approaches (Saltonstall et al., 2024). Within active filtration, Filter, and Sterivex behaved more similarly to each other than to Gauze, but they were not fully interchangeable. This distinction becomes important when studies aim to compare specific taxonomic groups, evenness-related diversity patterns or microbe-microbe associations, rather than only broad richness or community-level trends. Filter provided more stable richness estimates, whereas Sterivex tended to yield higher evenness-based diversity and greater relative representation of several protist groups. Taxonomically, Sterivex was associated with lower Alphaproteobacteria representation and higher Gammaproteobacteria and/or Bacteroidia representation in bacteria, as well as higher Syndiniales contribution and broader protist recovery in eukaryotes. The two active workflows also differed in the identity of some network hubs, indicating that workflow choice may influence conclusions about the ecological prominence or associations of particular taxa even when overall diversity patterns remain broadly similar. Such differences are plausible given the distinct architecture of the two active systems. Open flat filters require direct membrane handling and may be more sensitive to biomass redistribution and clogging, whereas enclosed Sterivex cartridges reduce field exposure but may impose constraints on biomass recovery and cell lysis (Patin and Goodwin, 2023). The significant differences observed here between Filter and Sterivex partly differ from (Pascoal et al. 2023), who reported broadly comparable richness between flat membrane and cartridge filters under controlled conditions. However, this difference should not be interpreted as evidence that either active method is universally more accurate. Rather, together with the stronger separation of Gauze, the results suggest that Filter and Sterivex are broadly comparable active-filtration approaches that can nevertheless produce context-dependent differences in diversity and composition.

The extent to which patterns of community structure were reproduced across sampling workflows differed between workflow pairs and microbial fractions. Filter and Sterivex reproduced microbial community structure for both bacterial and eukaryotic communities, consistent with previous methodological comparisons (Pascoal et al., 2023; Saltonstall et al., 2024; Qian et al., 2026), despite the differences observed in taxonomic recovery. Comparisons with Gauze suggested that it retained part of the same overall bacterial community pattern, although less close than the two active filtration workflows. For eukaryotic communities, Gauze reproduced eukaryotic community structure patterns less accurately than it did for bacterial communities, partly reflecting the taxonomic range retrieved by 18S metabarcoding, covering both protistan and metazoan signals (Anderson and Thompson, 2022; Dugal et al., 2023). Differences in the biological fractions retained by the workflows could therefore have a stronger influence on the resulting eukaryotic community configurations (Qian et al., 2026).

Sampling workflow was also associated with differences in inferred associations of both bacterial and eukaryotic communities, indicating that methodological effects extended beyond taxonomic composition to the organization of microbe-microbe interactions through co-occurrence networks. Because such networks are sensitive to compositionality, sparsity and data heterogeneity, the observed links are interpreted as statistical associations (Kurtz et al., 2015; Hirano and Takemoto, 2019; Matchado et al., 2021) and the present network analysis is considered exploratory given the limited sample size per network. Across bacterial networks, connectivity metrics varied among gulfs, with no single workflow producing the most connected network in all three locations. The only recurrent topological pattern was the highest clustering coefficient in Filter, which also produced the smallest network in all three gulfs. Since graph metrics can be affected by differences in network size and connectivity (van Wijk et al., 2010; Liu et al., 2023), this pattern cannot be interpreted biologically without a size-controlled comparison. Although OTU overlap was limited among workflows, Filter, and Sterivex shared highly connected OTUs in each gulf and recurrently included Alphaproteobacteria such as SAR116- and Rhodobacteraceae-related taxa. These lineages are common components of marine bacterioplankton communities (Simon et al., 2017; Šantić et al., 2021; Getz et al., 2023), suggesting some consistency in the bacterial groups emerging as highly connected under the two active-filtration workflows, despite local community differences. Gauze produced a largely distinct set of top hubs, including *Crocinitomix, Aurantivirga, Roseibacillus*, and members of OM60(NOR5), that belong to lineages previously associated with particle colonization or the utilization of algal and other high-molecular-weight organic matter (Oberbeckmann et al., 2016; Orsi et al., 2016; Krüger et al., 2019). Indeed, particulate material of various sizes can potentially be encountered by the passive, non-pre-sieved workflow (Kressler et al., 2026). For eukaryotes, Dinophyceae and Syndiniales formed recurrent taxonomic components across networks, suggesting that part of the eukaryotic association structure remained consistent despite differences in sampling workflow. The prominence of Syndiniales is ecologically plausible, given their widespread occurrence as marine parasitic alveolates and their recognized role in planktonic association networks (Christaki et al., 2017; Anderson et al., 2024). Similar results have also been reported from Aegean coastal systems, including the three gulfs examined here, where Dinophyceae and Syndiniales were prevalent and parasitic taxa contributed strongly to inferred protistan associations (Meziti et al., 2023). However, exact top-hub OTU overlap among workflows remained limited, while additional divergence was expressed in the secondary hub layer and in network architecture among gulfs. Notably, the two-node Kavala Gauze network further highlights that network inference was not equally informative across all workflow–location combinations. Network hubs and topological properties are often used to identify potentially influential taxa and interpret microbial community organization (Kurtz et al., 2015; Hirano and Takemoto, 2019). The observed ecological prominence and associations of individual taxa in such ecological analyses could be conditional on sampling workflow and should be evaluated cautiously across studies.

## 5 Conclusions

The findings of this study confirm that marine eDNA sampling workflows influence the bacterial and eukaryotic community signals recovered through metabarcoding. Filter and Sterivex produced the most concordant patterns of community structure and generally similar, although not fully equivalent, outputs. Sterivex's higher evenness-based representation of several protist groups, together with the Filter's greater richness stability, reflect some of their main discrepancies. Passive Gauze sampling, in contrast, produced variable and compositionally distinct signals, particularly for eukaryotic communities. This is consistent with differences in capture mechanisms and with the absence of the pre-sieving step in the Gauze workflow, which may partly contribute to the greater detection of particle- and organism-associated material. Gauze may therefore provide a complementary approach when broader biological material is of interest, albeit occasional low-yield recoveries and low replicability. Overall, workflow choice should be guided by the specific ecological question rather than by assuming a universally optimal approach, while maintaining a consistent workflow remains particularly important for monitoring and cross-study comparisons. Further work could strengthen the application of passive Gauze sampling by disentangling workflow components that were inherently coupled here. Applying Gauze with and without the 200-μm pre-screening used for the active workflows would help determine how much of its distinct signal reflects access to larger biological material, while replicated deployment-time experiments could establish how exposure duration affects recovery and variability. Replication in scaled settings would strengthen the mechanistic basis for selecting and standardizing fit-for-purpose eDNA sampling workflows.

## Data Availability

The datasets presented in this study can be found in online repositories. The names of the repository/repositories and accession number(s) can be found in the article/Supplementary material.
