# Supplementary material for: Environmental DNA sampling workflow matters: testing different approaches on marine microbial assemblages in three coastal areas of Greece
Source: Front Microbiol. 2026 Sep 25;17:1953819. doi: 10.3389/fmicb.2026.1953819 (PMC13650909; doi:10.3389/fmicb.2026.1953819)
Supplement: Supplementary file 1 [file Data_Sheet_1.docx]

Supplementary Material

# 1 Supplementary Figures and Tables

Table S1 Sampling metadata for all samples collected across the three study areas, including sampling site and code, sampling workflow, depth, processed water volume or deployment time, DNA yield, bacterial and eukaryotic read counts, geographic coordinates, and sampling date. Filter: active filtration using open membrane filters; Sterivex: active filtration using Sterivex cartridges; Gauze: passive sampling using gauze as the sorptive material.

| **Location** | **Sampling Site** | **Sampling point code** | **Method** | **Depth (m)** | **Filtered water volume** | **DNA yield (ng/μL)** | **Bacterial Read Counts** | **Eukaryotic Read Counts** | **Latitude** | **Longitude** | **Date** |
| --- | --- | --- | --- | --- | --- | --- | --- | --- | --- | --- | --- |
| Kavala | K1 | **F_K1s** | Filter | 0 | 1 L | 2.92 | 36,079 | 19,387 | 40.7291 | 24.2547 | 18/6/2024 |
|  |  | **G_K1s** | Gauze |  | 10 min | Low | 261 | 6 |  |  |  |
|  |  | **S_K1s** | Sterivex |  | 1 L | 9.25 | 18,620 | 800 |  |  |  |
|  |  | **F_K1d** | Filter | 40 | 1.5 L | 3.68 | 35,200 | 16,977 |  |  |  |
|  |  | **G_K1d** | Gauze |  | 10 min | Low | 1,475 | 115 |  |  |  |
|  |  | **S_K1d** | Sterivex |  | 1.5 L | 7.23 | 19,756 | 471 |  |  |  |
|  | K2 | **F_K2s** | Filter | 0 | 1 L | 4.58 | 44,946 | 16,102 | 40.731 | 24.2116 | 18/6/2024 |
|  |  | **G_K2s** | Gauze |  | 10 min | Low | 4,555 | 298 |  |  |  |
|  |  | **S_K2s** | Sterivex |  | 1 L | 7.78 | 19,227 | 659 |  |  |  |
|  |  | **F_K2d** | Filter | 40 | 1.5 L | 4.56 | 47,196 | 17,815 |  |  |  |
|  |  | **G_K2d** | Gauze |  | 10 min | Low | 13,695 | 451 |  |  |  |
|  |  | **S_K2d** | Sterivex |  | 1.5 L | 4.37 | 18,701 | 2,108 |  |  |  |
|  | K3 | **F_K3s** | Filter | 0 | 1 L | 6.66 | 46,267 | 12,959 | 40.7722 | 24.3521 | 18/6/2024 |
|  |  | **G_K3s** | Gauze |  | 10 min | Low | 9,252 | 47 |  |  |  |
|  |  | **S_K3s** | Sterivex |  | 1 L | 5.91 | 18,974 | 749 |  |  |  |
|  |  | **F_K3d** | Filter | 20 | 1.3 L | 3.42 | 37,124 | 15,093 |  |  |  |
|  |  | **G_K3d** | Gauze |  | 10 min | Low | 8,894 | 167 |  |  |  |
|  |  | **S_K3d** | Sterivex |  | 1.3 L | 12.80 | 20,967 | 6,696 |  |  |  |
|  | K4 | **F_K4s** | Filter | 0 | 1.1 L | 3.38 | 33,939 | 8,783 | 40.817 | 24.4126 | 18/6/2024 |
|  |  | **G_K4s** | Gauze |  | 10 min | Low | 167 | 1 |  |  |  |
|  |  | **S_K4s** | Sterivex |  | 1.1 L | 8.27 | 21,348 | 28,135 |  |  |  |
|  |  | **F_K4d** | Filter | 20 | 1.3 L | 9 | 31,698 | 16,982 |  |  |  |
|  |  | **G_K4d** | Gauze |  | 10 min | Low | 72 | 1 |  |  |  |
|  |  | **S_K4d** | Sterivex |  | 1.3 L | 11.67 | 17,934 | 2,144 |  |  |  |
|  | K5 | **F_K5s** | Filter | 0 | 1 L | 15.1 | 39,480 | 14,683 | 40.836 | 24.3559 | 18/6/2024 |
|  |  | **G_K5s** | Gauze |  | 10 min | Low | 20,552 | 2,075 |  |  |  |
|  |  | **S_K5s** | Sterivex |  | 1 L | 9.63 | 16,933 | 5,774 |  |  |  |
|  |  | **F_K5d** | Filter | 12 | 1.3 L | 13.8 | 39,551 | 31,769 |  |  |  |
|  |  | **G_K5d** | Gauze |  | 10 min | Low | 15,448 | 27 |  |  |  |
|  |  | **S_K5d** | Sterivex |  | 1.3 L | 13.57 | 23,161 | 12,903 |  |  |  |
|  | K6 | **F_K6s** | Filter | 0 | 1 L | 10.2 | 47,759 | 18,370 | 40.838 | 24.3212 | 18/6/2024 |
|  |  | **G_K6s** | Gauze |  | 10 min | Low | 15,516 | 282 |  |  |  |
|  |  | **S_K6s** | Sterivex |  | 1 L | 11.67 | 4,459 | 7,180 |  |  |  |
|  |  | **F_K6d** | Filter | 11 | 1.3 L | 13 | 41,229 | 13,958 |  |  |  |
|  |  | **G_K6d** | Gauze |  | 10 min | Low | 11,190 | 54 |  |  |  |
|  |  | **S_K6d** | Sterivex |  | 1.3 L | 8.85 | 24,515 | 4,278 |  |  |  |
| Saronikos | S1 | **F_S1s** | Filter | 1 | 1 L | 17.9 | 42,503 | 12,755 | 37.9929 | 23.5482 | 22/4/2024 |
|  |  | **G_S1s** | Gauze |  | 10 min | Low | 16,584 | 53 |  |  |  |
|  |  | **S_S1s** | Sterivex |  | 1 L | 2.41 | 16,671 | 311 |  |  |  |
|  |  | **F_S1d** | Filter | 20 | 1.4 L | 58.6 | 28,399 | 16,130 |  |  |  |
|  |  | **G_S1d** | Gauze |  | 10 min | 0.25 | 7,530 | 581 |  |  |  |
|  |  | **S_S1d** | Sterivex |  | 1.4 L | 2.58 | 20,501 | 381 |  |  |  |
|  | S2 | **F_S2s** | Filter | 1 | 1.1 L | 9.56 | 55,955 | 8,343 | 37.8979 | 23.5471 | 22/4/2024 |
|  |  | **G_S2s** | Gauze |  | 10 min | 0.22 | 11,578 | 402 |  |  |  |
|  |  | **S_S2s** | Sterivex |  | 1.1 L | 0.98 | 19,369 | 142 |  |  |  |
|  |  | **F_S2d** | Filter | 30 | 1.5 L | 3.78 | 56,358 | 11,529 |  |  |  |
|  |  | **G_S2d** | Gauze |  | 10 min | 0.18 | 10,716 | 126 |  |  |  |
|  |  | **S_S2d** | Sterivex |  | 1.5 L | 3.32 | 36,670 | 150 |  |  |  |
|  | S3 | **F_S3s** | Filter | 1 | 1 L | 13.7 | 43,940 | 8,511 | 37.8503 | 23.433 | 22/4/2024 |
|  |  | **G_S3s** | Gauze |  | 10 min | 0.12 | 98,172 | 4,729 |  |  |  |
|  |  | **S_S3s** | Sterivex |  | 1 L | 3.79 | 26,217 | 380 |  |  |  |
|  |  | **F_S3d** | Filter | 30 | 1.5 L | 4.62 | 40,357 | 16,620 |  |  |  |
|  |  | **G_S3d** | Gauze |  | 10 min | Low | 1,722 | 1,725 |  |  |  |
|  |  | **S_S3d** | Sterivex |  | 1.5 L | 5.85 | 26,636 | 928 |  |  |  |
|  | S4 | **F_S4s** | Filter | 1 | 1 L | 15.9 | 39,691 | 11,653 | 37.9028 | 23.3803 | 22/4/2024 |
|  |  | **G_S4s** | Gauze |  | 10 min | 0.10 | 13,926 | 301 |  |  |  |
|  |  | **S_S4s** | Sterivex |  | 1 L | 3.69 | 33,221 | 511 |  |  |  |
|  |  | **F_S4d** | Filter | 30 | 1.5 L | 21 | 46,355 | 15,115 |  |  |  |
|  |  | **G_S4d** | Gauze |  | 10 min | Low | 11,007 | 2,934 |  |  |  |
|  |  | **S_S4d** | Sterivex |  | 1.5 L | 6.13 | 9,623 | 1,806 |  |  |  |
|  | S5 | **F_S5s** | Filter | 1 | 1 L | 15 | 37,393 | 15,027 | 37.981 | 23.4139 | 22/4/2024 |
|  |  | **G_S5s** | Gauze |  | 10 min | Low | 201,213 | 2,969 |  |  |  |
|  |  | **S_S5s** | Sterivex |  | 1 L | 7.17 | 20,890 | 1,920 |  |  |  |
|  |  | **F_S5d** | Filter | 10 | 1.3 L | 2.98 | 42,129 | 11,084 |  |  |  |
|  |  | **G_S5d** | Gauze |  | 10 min | Low | 1,138 | 285 |  |  |  |
|  |  | **S_S5d** | Sterivex |  | 1.3 L | 5.10 | 27,897 | 322 |  |  |  |
|  | S6 | **F_S6s** | Filter | 1 | 1.1 L | 10.36 | 27,955 | 18,013 | 38.0132 | 23.4955 | 22/4/2024 |
|  |  | **G_S6s** | Gauze |  | 10 min | 0.12 | 9,661 | 10,007 |  |  |  |
|  |  | **S_S6s** | Sterivex |  | 1.1 L | 6.83 | 40,544 | 12,269 |  |  |  |
|  |  | **F_S6d** | Filter | 14 | 1.3 L | 7.5 | 36,775 | 37,197 |  |  |  |
|  |  | **G_S6d** | Gauze |  | 10 min | 0.38 | 361,417 | 7,552 |  |  |  |
|  |  | **S_S6d** | Sterivex |  | 1.3 L | 1.71 | 36,953 | 1,090 |  |  |  |
| Thermaikos | T1 | **F_T1s** | Filter | 0 | 1 L | 20 | 30,880 | 40,662 | 40.5469 | 22.7625 | 16/6/2024 |
|  |  | **G_T1s** | Gauze |  | 10 min | 0.49 | 15,265 | 13,644 |  |  |  |
|  |  | **S_T1s** | Sterivex |  | 1 L | 51.40 | 25,980 | 31,144 |  |  |  |
|  |  | **F_T1d** | Filter | 18 | 1.5 L | 17.6 | 31,299 | 23,865 |  |  |  |
|  |  | **G_T1d** | Gauze |  | 10 min | 1.00 | 18,159 | 23,827 |  |  |  |
|  |  | **S_T1d** | Sterivex |  | 1.5 L | 16.33 | 19,033 | 19,115 |  |  |  |
|  | T2 | **F_T2s** | Filter | 0 | 1 L | 23.4 | 34,274 | 23,486 | 40.6209 | 22.8958 | 16/6/2024 |
|  |  | **G_T2s** | Gauze |  | 10 min | 2.81 | 18,671 | 30,815 |  |  |  |
|  |  | **S_T2s** | Sterivex |  | 1 L | 81.60 | 13,751 | 15,422 |  |  |  |
|  |  | **F_T2d** | Filter | 12 | 1.4 L | 28.2 | 29,580 | 20,327 |  |  |  |
|  |  | **G_T2d** | Gauze |  | 10 min | 0.94 | 19,242 | 31,480 |  |  |  |
|  |  | **S_T2d** | Sterivex |  | 1.4 L | 71.60 | 2,490 | 29,932 |  |  |  |
|  | T3 | **F_T3s** | Filter | 0 | 1.1 L | 21.8 | 32,693 | 23,282 | 40.6248 | 22.938 | 16/6/2024 |
|  |  | **G_T3s** | Gauze |  | 10 min | 0.89 | 2 | 5,858 |  |  |  |
|  |  | **S_T3s** | Sterivex |  | 1.1 L | 78.09 | 15,968 | 17,074 |  |  |  |
|  |  | **F_T3d** | Filter | 11 | 1.5 L | 21.4 | 28,802 | 16,157 |  |  |  |
|  |  | **G_T3d** | Gauze |  | 10 min | 0.77 | 30,602 | 3,653 |  |  |  |
|  |  | **S_T3d** | Sterivex |  | 1.5 L | 24.93 | 16,206 | 344 |  |  |  |
|  | T4 | **F_T4s** | Filter | 0 | 1.1 L | 16.9 | 34,031 | 25,393 | 40.5652 | 22.954 | 16/6/2024 |
|  |  | **G_T4s** | Gauze |  | 10 min | 0.96 | 22,058 | 776 |  |  |  |
|  |  | **S_T4s** | Sterivex |  | 1.1 L | 34.50 | 37,227 | 6,971 |  |  |  |
|  |  | **F_T4d** | Filter | 7 | 1.4 L | 24 | 34,421 | 29,591 |  |  |  |
|  |  | **G_T4d** | Gauze |  | 10 min | 5.93 | 10,353 | 5,247 |  |  |  |
|  |  | **S_T4d** | Sterivex |  | 1.4 L | 38.28 | 27,112 | 2,484 |  |  |  |
|  | T5 | **F_T5s** | Filter | 0 | 1 L | 20.6 | 32,068 | 43,093 | 40.5139 | 22.8481 | 16/6/2024 |
|  |  | **G_T5s** | Gauze |  | 10 min | 0.16 | 217 | NA |  |  |  |
|  |  | **S_T5s** | Sterivex |  | 1 L | 15.53 | 14,999 | 75,555 |  |  |  |
|  |  | **F_T5d** | Filter | 20 | 1.5 L | 37.8 | 26,422 | 11,724 |  |  |  |
|  |  | **G_T5d** | Gauze |  | 10 min | 0.48 | 16,291 | 3,496 |  |  |  |
|  |  | **S_T5d** | Sterivex |  | 1.5 L | 9.20 | 18,846 | 8,876 |  |  |  |
|  | T6 | **F_T6s** | Filter | 0 | 1.1 L | 4.34 | 32,208 | 16,370 | 40.3729 | 22.9285 | 16/6/2024 |
|  |  | **G_T6s** | Gauze |  | 10 min | 0.13 | 11,988 | 1,105 |  |  |  |
|  |  | **S_T6s** | Sterivex |  | 1.1 L | 9.82 | 18,774 | 17,229 |  |  |  |
|  |  | **F_T6d** | Filter | 20 | 1.5 L | 25.6 | 42,313 | 15,650 |  |  |  |
|  |  | **G_T6d** | Gauze |  | 10 min | 0.18 | 16,031 | 1,784 |  |  |  |
|  |  | **S_T6d** | Sterivex |  | 1.5 L | 6.29 | 20,741 | 17,098 |  |  |  |

Table S2 Model diagnostics and pairwise effect estimates for bacterial and eukaryotic alpha-diversity models. Model convergence was assessed from optimizer convergence and Hessian diagnostics, and residual adequacy was evaluated using DHARMa simulation-based tests of uniformity, dispersion and outliers (5,000 simulations). Pairwise contrasts were calculated from estimated marginal means and are reported as back-transformed ratios for negative-binomial and Gamma models and odds ratios for beta models, with asymptotic 95% confidence intervals (CI). Pairwise p-values were adjusted within each alpha-diversity index using the Benjamini–Hochberg procedure.

| **Group** | **Alpha index** | **Converged** | **DHARMa uniformity p** | **Dispersion p** | **Outlier p** | **Contrast** | **Effect** | **Estimate** | **95% CI** | **BH-adjusted p** |
| --- | --- | --- | --- | --- | --- | --- | --- | --- | --- | --- |
| Bacteria | OTU richness | Yes | 0.160 | 0.736 | 1.000 | Filter / Gauze | Ratio | 0.710 | 0.535–0.944 | 0.027 |
|  |  |  |  |  |  | Filter / Sterivex | Ratio | 1.079 | 0.998–1.167 | 0.057 |
|  |  |  |  |  |  | Gauze / Sterivex | Ratio | 1.519 | 1.133–2.037 | 0.015 |
| Bacteria | Shannon | Yes | 0.716 | 0.046 | 0.042 | Filter / Gauze | Ratio | 0.810 | 0.736–0.892 | <0.001 |
|  |  |  |  |  |  | Filter / Sterivex | Ratio | 0.881 | 0.859–0.904 | <0.001 |
|  |  |  |  |  |  | Gauze / Sterivex | Ratio | 1.088 | 0.988–1.197 | 0.085 |
| Bacteria | Simpson | Yes | 0.249 | 0.042 | 0.042 | Filter / Gauze | Odds ratio | 0.711 | 0.517–0.978 | 0.054 |
|  |  |  |  |  |  | Filter / Sterivex | Odds ratio | 0.603 | 0.546–0.666 | <0.001 |
|  |  |  |  |  |  | Gauze / Sterivex | Odds ratio | 0.849 | 0.612–1.178 | 0.327 |
| Bacteria | Evenness | Yes | 0.116 | 0.214 | 0.042 | Filter / Gauze | Odds ratio | 0.480 | 0.394–0.586 | <0.001 |
|  |  |  |  |  |  | Filter / Sterivex | Odds ratio | 0.678 | 0.646–0.713 | <0.001 |
|  |  |  |  |  |  | Gauze / Sterivex | Odds ratio | 1.412 | 1.161–1.717 | <0.001 |
| Eukaryotes | OTU richness | Yes | 0.356 | 0.099 | 1.000 | Filter / Gauze | Ratio | 1.906 | 1.35–2.69 | <0.001 |
|  |  |  |  |  |  | Filter / Sterivex | Ratio | 0.984 | 0.704–1.37 | 0.923 |
|  |  |  |  |  |  | Gauze / Sterivex | Ratio | 0.516 | 0.366–0.727 | <0.001 |
| Eukaryotes | Shannon | Yes | 0.006 | 0.049 | 1.000 | Filter / Gauze | Ratio | 0.926 | 0.770–1.11 | 0.409 |
|  |  |  |  |  |  | Filter / Sterivex | Ratio | 0.709 | 0.592–0.848 | <0.001 |
|  |  |  |  |  |  | Gauze / Sterivex | Ratio | 0.766 | 0.637–0.920 | 0.007 |
| Eukaryotes | Simpson | Yes | 0.010 | 0.156 | 1.000 | Filter / Gauze | Odds ratio | 0.734 | 0.507–1.06 | 0.100 |
|  |  |  |  |  |  | Filter / Sterivex | Odds ratio | 0.511 | 0.353–0.742 | 0.001 |
|  |  |  |  |  |  | Gauze / Sterivex | Odds ratio | 0.697 | 0.474–1.02 | 0.099 |
| Eukaryotes | Evenness | Yes | 0.166 | 0.930 | 1.000 | Filter / Gauze | Odds ratio | 0.517 | 0.393–0.680 | <0.001 |
|  |  |  |  |  |  | Filter / Sterivex | Odds ratio | 0.448 | 0.342–0.587 | <0.001 |
|  |  |  |  |  |  | Gauze / Sterivex | Odds ratio | 0.867 | 0.660–1.14 | 0.305 |

Table S3 Retained OTUs, pairwise OTU comparisons, and associations retained for each co-occurrence network after Benjamini–Hochberg correction and application of the MIC threshold.

|  | **BACTERIA** | | | | | | | | |
| --- | --- | --- | --- | --- | --- | --- | --- | --- | --- |
|  | **Kavala** | | | **Saronikos** | | | **Thermaikos** | | |
|  | **Filter** | **Gauze** | **Sterivex** | **Filter** | **Gauze** | **Sterivex** | **Filter** | **Gauze** | **Sterivex** |
| **Retained OTUs** | 71 | 138 | 85 | 83 | 117 | 89 | 107 | 135 | 126 |
| **OTU pairs tested** | 2485 | 9453 | 3570 | 3403 | 6786 | 3916 | 5671 | 9045 | 7875 |
| **Retained associations (network edges)** | 729 | 1850 | 721 | 782 | 3014 | 534 | 29 | 71 | 527 |
|  | **EUKARYOTES** | | | | | | | | |
|  | **Kavala** | | | **Saronikos** | | | **Thermaikos** | | |
|  | **Filter** | **Gauze** | **Sterivex** | **Filter** | **Gauze** | **Sterivex** | **Filter** | **Gauze** | **Sterivex** |
| **Retained OTUs** | 59 | 58 | 152 | 74 | 72 | 83 | 54 | 77 | 92 |
| **OTU pairs tested** | 1711 | 1653 | 11476 | 2701 | 2556 | 3403 | 1431 | 2926 | 4186 |
| **Retained associations (network edges)** | 7 | 1 | 59 | 28 | 56 | 15 | 12 | 386 | 203 |

Table S4 Alpha-diversity indices of the bacterial and eukaryotic communities in each sample. Empty cells indicate samples for which alpha-diversity indices were not calculated because the corresponding sample yielded insufficient reads after quality filtering. See Supplementary Table S1 for sampling code annotations.

|  | BACTERIA | | | | EUKARYOTES | | | |
| --- | --- | --- | --- | --- | --- | --- | --- | --- |
| Sampling points | OTUs richness | Shannon (H) | Simpson (1-D) | Pielou’s J | OTUs richness | Shannon (H) | Simpson (1-D) | Pielou’s J |
| F_K1d | 275 | 3.064 | 0.879 | 0.546 | 183 | 1.937 | 0.647 | 0.372 |
| F_K1s | 174 | 2.908 | 0.880 | 0.564 | 163 | 2.137 | 0.704 | 0.419 |
| F_K2d | 275 | 2.582 | 0.805 | 0.460 | 206 | 1.912 | 0.570 | 0.359 |
| F_K2s | 189 | 2.795 | 0.875 | 0.533 | 208 | 2.507 | 0.742 | 0.470 |
| F_K3d | 237 | 3.128 | 0.893 | 0.572 | 201 | 2.147 | 0.688 | 0.405 |
| F_K3s | 167 | 2.787 | 0.876 | 0.544 | 184 | 2.553 | 0.772 | 0.490 |
| F_K4d | 227 | 3.101 | 0.889 | 0.572 | 144 | 2.251 | 0.806 | 0.453 |
| F_K4s | 159 | 2.852 | 0.886 | 0.563 | 141 | 2.402 | 0.811 | 0.485 |
| F_K5d | 260 | 3.076 | 0.892 | 0.553 | 200 | 1.168 | 0.556 | 0.220 |
| F_K5s | 192 | 3.064 | 0.879 | 0.546 | 184 | 1.784 | 0.577 | 0.342 |
| F_K6d | 247 | 2.908 | 0.880 | 0.564 | 223 | 2.492 | 0.803 | 0.461 |
| F_K6s | 212 | 2.582 | 0.805 | 0.460 | 166 | 2.083 | 0.713 | 0.408 |
| F_S1d | 301 | 2.795 | 0.875 | 0.533 | 107 | 0.575 | 0.173 | 0.123 |
| F_S1s | 209 | 3.128 | 0.893 | 0.572 | 126 | 2.326 | 0.758 | 0.481 |
| F_S2d | 246 | 2.787 | 0.876 | 0.544 | 194 | 2.372 | 0.792 | 0.450 |
| F_S2s | 240 | 3.101 | 0.889 | 0.572 | 232 | 3.077 | 0.826 | 0.565 |
| F_S3d | 241 | 2.852 | 0.886 | 0.563 | 270 | 2.525 | 0.718 | 0.451 |
| F_S3s | 250 | 3.076 | 0.892 | 0.553 | 194 | 2.743 | 0.799 | 0.521 |
| F_S4d | 257 | 3.064 | 0.879 | 0.546 | 217 | 2.663 | 0.840 | 0.495 |
| F_S4s | 242 | 2.908 | 0.880 | 0.564 | 207 | 3.158 | 0.901 | 0.592 |
| F_S5d | 268 | 2.582 | 0.805 | 0.460 | 175 | 2.714 | 0.861 | 0.526 |
| F_S5s | 234 | 3.345 | 0.927 | 0.613 | 198 | 2.651 | 0.843 | 0.501 |
| F_S6d | 247 | 3.349 | 0.935 | 0.608 | 115 | 0.783 | 0.278 | 0.165 |
| F_S6s | 202 | 3.158 | 0.916 | 0.595 | 151 | 2.294 | 0.800 | 0.457 |
| F_T1d | 329 | 3.932 | 0.956 | 0.678 | 159 | 1.085 | 0.331 | 0.214 |
| F_T1s | 277 | 3.206 | 0.903 | 0.570 | 185 | 1.560 | 0.631 | 0.299 |
| F_T2d | 351 | 3.896 | 0.935 | 0.665 | 142 | 1.681 | 0.630 | 0.339 |
| F_T2s | 290 | 3.613 | 0.935 | 0.637 | 157 | 1.765 | 0.629 | 0.349 |
| F_T3d | 324 | 3.783 | 0.923 | 0.654 | 217 | 2.481 | 0.798 | 0.461 |
| F_T3s | 288 | 3.728 | 0.950 | 0.658 | 128 | 1.506 | 0.691 | 0.310 |
| F_T4d | 291 | 3.467 | 0.936 | 0.611 | 162 | 0.965 | 0.322 | 0.190 |
| F_T4s | 257 | 3.166 | 0.906 | 0.570 | 184 | 1.835 | 0.647 | 0.352 |
| F_T5d | 307 | 3.555 | 0.911 | 0.621 | 140 | 1.986 | 0.660 | 0.402 |
| F_T5s | 256 | 3.183 | 0.910 | 0.574 | 97 | 0.625 | 0.271 | 0.137 |
| F_T6d | 269 | 3.061 | 0.888 | 0.547 | 233 | 2.056 | 0.595 | 0.377 |
| F_T6s | 189 | 3.092 | 0.907 | 0.590 | 212 | 2.328 | 0.703 | 0.435 |
| G_K1s | 49 | 3.354 | 0.950 | 0.862 | 4 | 1.242 | 0.667 | 0.896 |
| G_K1d | 147 | 4.317 | 0.981 | 0.865 | 7 | 0.598 | 0.240 | 0.307 |
| G_K2s | 179 | 4.008 | 0.968 | 0.773 | 42 | 2.376 | 0.827 | 0.636 |
| G_K2d | 315 | 3.973 | 0.952 | 0.691 | 29 | 1.652 | 0.707 | 0.490 |
| G_K3s | 180 | 4.024 | 0.968 | 0.775 | 15 | 1.882 | 0.745 | 0.695 |
| G_K3d | 238 | 4.028 | 0.963 | 0.736 | 30 | 2.477 | 0.852 | 0.728 |
| G_K4s | 29 | 3.038 | 0.941 | 0.902 |  |  |  |  |
| G_K4d | 19 | 2.396 | 0.874 | 0.814 |  |  |  |  |
| G_K5s | 325 | 4.402 | 0.975 | 0.761 | 63 | 1.225 | 0.394 | 0.296 |
| G_K5d | 382 | 4.447 | 0.972 | 0.748 | 10 | 1.845 | 0.760 | 0.801 |
| G_K6s | 304 | 4.040 | 0.946 | 0.707 | 19 | 1.184 | 0.471 | 0.402 |
| G_K6d | 348 | 4.450 | 0.977 | 0.760 | 16 | 2.236 | 0.846 | 0.806 |
| G_S1s | 287 | 4.247 | 0.970 | 0.750 | 19 | 2.461 | 0.878 | 0.836 |
| G_S1d | 329 | 4.626 | 0.982 | 0.798 | 65 | 3.072 | 0.924 | 0.736 |
| G_S2s | 198 | 2.351 | 0.692 | 0.445 | 37 | 2.035 | 0.723 | 0.564 |
| G_S2d | 195 | 2.665 | 0.747 | 0.505 | 8 | 0.473 | 0.178 | 0.228 |
| G_S3s | 510 | 3.845 | 0.932 | 0.617 | 74 | 0.486 | 0.149 | 0.113 |
| G_S3d | 131 | 3.573 | 0.935 | 0.733 | 113 | 2.613 | 0.793 | 0.553 |
| G_S4s | 265 | 3.854 | 0.938 | 0.691 | 54 | 2.982 | 0.891 | 0.748 |
| G_S4d | 276 | 4.034 | 0.952 | 0.718 | 130 | 2.508 | 0.767 | 0.515 |
| G_S5s | 1832 | 4.835 | 0.977 | 0.644 | 148 | 3.174 | 0.833 | 0.635 |
| G_S5d | 115 | 4.033 | 0.971 | 0.850 | 17 | 0.951 | 0.395 | 0.336 |
| G_S6s | 268 | 4.204 | 0.972 | 0.752 | 109 | 1.777 | 0.702 | 0.379 |
| G_S6d | 929 | 4.791 | 0.984 | 0.701 | 145 | 2.687 | 0.817 | 0.540 |
| G_T1s | 458 | 4.211 | 0.958 | 0.687 | 250 | 3.456 | 0.907 | 0.626 |
| G_T1d | 523 | 4.288 | 0.936 | 0.685 | 284 | 2.799 | 0.871 | 0.495 |
| G_T2s | 446 | 4.442 | 0.975 | 0.728 | 242 | 2.854 | 0.879 | 0.520 |
| G_T2d | 499 | 4.790 | 0.980 | 0.771 | 287 | 2.768 | 0.837 | 0.489 |
| G_T3s | 2 | 0.693 | 0.500 | 1.000 | 166 | 2.861 | 0.886 | 0.560 |
| G_T3d | 584 | 4.800 | 0.981 | 0.754 | 159 | 3.048 | 0.905 | 0.601 |
| G_T4s | 464 | 4.156 | 0.957 | 0.677 | 69 | 2.493 | 0.812 | 0.589 |
| G_T4d | 722 | 5.167 | 0.982 | 0.785 | 148 | 2.793 | 0.862 | 0.559 |
| G_T5s | 27 | 2.811 | 0.917 | 0.853 |  |  |  |  |
| G_T5d | 439 | 4.180 | 0.947 | 0.687 | 109 | 2.294 | 0.819 | 0.489 |
| G_T6s | 375 | 4.245 | 0.966 | 0.716 | 54 | 2.077 | 0.749 | 0.521 |
| G_T6d | 437 | 4.149 | 0.937 | 0.682 | 104 | 2.887 | 0.907 | 0.622 |
| S_K1s | 131 | 3.341 | 0.938 | 0.685 | 102 | 3.175 | 0.881 | 0.686 |
| S_K1d | 237 | 3.491 | 0.922 | 0.638 | 101 | 3.256 | 0.869 | 0.705 |
| S_K2s | 149 | 3.363 | 0.938 | 0.672 | 119 | 3.571 | 0.905 | 0.747 |
| S_K2d | 272 | 3.233 | 0.826 | 0.577 | 164 | 3.278 | 0.884 | 0.643 |
| S_K3s | 132 | 3.365 | 0.941 | 0.689 | 128 | 3.752 | 0.942 | 0.773 |
| S_K3d | 206 | 3.491 | 0.933 | 0.655 | 268 | 2.936 | 0.816 | 0.525 |
| S_K4s | 132 | 3.316 | 0.936 | 0.679 | 375 | 4.171 | 0.946 | 0.704 |
| S_K4d | 178 | 3.480 | 0.937 | 0.672 | 216 | 3.544 | 0.878 | 0.659 |
| S_K5s | 174 | 3.420 | 0.932 | 0.663 | 264 | 3.804 | 0.896 | 0.682 |
| S_K5d | 276 | 3.681 | 0.947 | 0.655 | 494 | 4.183 | 0.908 | 0.674 |
| S_K6s | 110 | 3.179 | 0.912 | 0.676 | 239 | 3.148 | 0.792 | 0.575 |
| S_K6d | 246 | 3.579 | 0.932 | 0.650 | 272 | 4.024 | 0.938 | 0.718 |
| S_S1s | 168 | 3.594 | 0.954 | 0.701 | 19 | 1.778 | 0.746 | 0.604 |
| S_S1d | 321 | 3.996 | 0.968 | 0.692 | 26 | 1.134 | 0.398 | 0.348 |
| S_S2s | 202 | 3.613 | 0.942 | 0.681 | 61 | 3.422 | 0.933 | 0.832 |
| S_S2d | 232 | 3.401 | 0.927 | 0.624 | 46 | 3.191 | 0.927 | 0.834 |
| S_S3s | 203 | 3.530 | 0.930 | 0.664 | 28 | 2.032 | 0.796 | 0.610 |
| S_S3d | 241 | 3.436 | 0.921 | 0.626 | 111 | 3.046 | 0.887 | 0.647 |
| S_S4s | 233 | 3.667 | 0.943 | 0.673 | 27 | 2.178 | 0.828 | 0.661 |
| S_S4d | 183 | 3.483 | 0.888 | 0.669 | 103 | 3.214 | 0.923 | 0.694 |
| S_S5s | 262 | 3.509 | 0.932 | 0.630 | 119 | 2.875 | 0.859 | 0.602 |
| S_S5d | 247 | 3.664 | 0.947 | 0.665 | 40 | 2.199 | 0.776 | 0.596 |
| S_S6s | 250 | 3.466 | 0.940 | 0.628 | 91 | 1.794 | 0.669 | 0.398 |
| S_S6d | 218 | 3.448 | 0.943 | 0.640 | 30 | 1.613 | 0.667 | 0.474 |
| S_T1s | 324 | 3.817 | 0.950 | 0.660 | 342 | 3.350 | 0.879 | 0.574 |
| S_T1d | 331 | 4.217 | 0.972 | 0.727 | 180 | 2.292 | 0.782 | 0.441 |
| S_T2s | 260 | 3.814 | 0.939 | 0.686 | 174 | 1.503 | 0.494 | 0.291 |
| S_T2d | 101 | 3.120 | 0.918 | 0.676 | 356 | 3.530 | 0.903 | 0.601 |
| S_T3s | 293 | 4.043 | 0.961 | 0.712 | 154 | 1.187 | 0.402 | 0.236 |
| S_T3d | 361 | 4.531 | 0.979 | 0.769 | 29 | 2.042 | 0.769 | 0.606 |
| S_T4s | 323 | 3.798 | 0.950 | 0.657 | 179 | 2.127 | 0.594 | 0.410 |
| S_T4d | 328 | 3.920 | 0.957 | 0.677 | 122 | 2.059 | 0.607 | 0.429 |
| S_T5s | 256 | 4.028 | 0.964 | 0.726 | 373 | 2.306 | 0.632 | 0.389 |
| S_T5d | 317 | 4.220 | 0.975 | 0.733 | 238 | 3.150 | 0.861 | 0.576 |
| S_T6s | 191 | 3.578 | 0.951 | 0.681 | 416 | 3.983 | 0.918 | 0.660 |
| S_T6d | 272 | 3.688 | 0.926 | 0.658 | 401 | 3.811 | 0.935 | 0.636 |

Table S5 Up to 10 OTU network hubs with the highest number of connections for each method and gulf. When fewer than 10 nodes were present in a network, all available nodes are reported.

| **BACTERIA** | | | | | |
| --- | --- | --- | --- | --- | --- |
| **Location** | **Method** | **OTU** | **Connections** | **Class** | **Genus** |
| Kavala | Filter | Otu0010 | 45 | Alphaproteobacteria | Candidatus *Puniceispirillum* |
|  |  | Otu0021 | 45 | Alphaproteobacteria | S25-593 |
|  |  | Otu0174 | 42 | Gammaproteobacteria | Unclassified Halieaceae |
|  |  | Otu0082 | 37 | Alphaproteobacteria | SAR116 clade |
|  |  | Otu0110 | 37 | Gammaproteobacteria | SAR86 clade |
|  |  | Otu0025 | 35 | Bacteroidia | Uncultured |
|  |  | Otu0079 | 35 | Alphaproteobacteria | Clade III |
|  |  | Otu0081 | 35 | Alphaproteobacteria | Clade IV |
|  |  | Otu0094 | 35 | Gammaproteobacteria | SAR92 clade |
|  |  | Otu0106 | 35 | Alphaproteobacteria | Unclassified Rhodobacteraceae |
|  | Gauze | Otu0023 | 77 | Gammaproteobacteria | OM60(NOR5) clade |
|  |  | Otu0032 | 76 | Bacteroidia | NS4 marine group |
|  |  | Otu0044 | 76 | Alphaproteobacteria | Unclassified Alphaproteobacteria |
|  |  | Otu0139 | 72 | Gammaproteobacteria | SAR86 clade |
|  |  | Otu0002 | 71 | Cyanobacteria | Unclassified Cyanobiaceae |
|  |  | Otu0036 | 66 | Alphaproteobacteria | AEGEAN-169 marine group |
|  |  | Otu0097 | 64 | OM190 | OM190 |
|  |  | Otu0158 | 63 | Bdellovibrionia | OM27 clade |
|  |  | Otu0071 | 57 | Bacteroidia | NS2b marine group |
|  |  | Otu0003 | 56 | Alphaproteobacteria | AEGEAN-169 marine group |
|  | Sterivex | Otu0079 | 43 | Alphaproteobacteria | Clade III |
|  |  | Otu0042 | 41 | Bacteroidia | *Formosa* |
|  |  | Otu0021 | 39 | Alphaproteobacteria | S25 593 |
|  |  | Otu0102 | 33 | Alphaproteobacteria | Clade IV |
|  |  | Otu0118 | 33 | Gammaproteobacteria | Uncultured |
|  |  | Otu0125 | 33 | Alphaproteobacteria | OM75 clade |
|  |  | Otu0146 | 33 | Gammaproteobacteria | SAR86 clade |
|  |  | Otu0150 | 33 | Alphaproteobacteria | Unclassified Rhodobacteraceae |
|  |  | Otu0152 | 33 | Alphaproteobacteria | SAR116 clade |
|  |  | Otu0006 | 32 | Cyanobacteria | *Prochlorococcus* MIT9313 |
| Saronikos | Filter | Otu0009 | 43 | Verrucomicrobiae | *Lentimonas* |
|  |  | Otu0015 | 43 | Alphaproteobacteria | SAR116 clade |
|  |  | Otu0001 | 42 | Alphaproteobacteria | Clade Ia |
|  |  | Otu0102 | 42 | Alphaproteobacteria | Clade IV |
|  |  | Otu0048 | 41 | Alphaproteobacteria | Clade IV |
|  |  | Otu0006 | 40 | Cyanobacteria | *Prochlorococcus* MIT9313 |
|  |  | Otu0032 | 40 | Bacteroidia | NS4 marine group |
|  |  | Otu0056 | 40 | Kiritimatiellae | R76-B128 |
|  |  | Otu0091 | 40 | Alphaproteobacteria | Unclassified Rhodobacteraceae |
|  |  | Otu0055 | 39 | Planctomycetes | Unclassified Pirellulaceae |
|  | Gauze | Otu0094 | 87 | Gammaproteobacteria | SAR92 clade |
|  |  | Otu0092 | 85 | Bacteroidia | NS7 marine group |
|  |  | Otu0225 | 84 | Bacteroidia | *Crocinitomix* |
|  |  | Otu0060 | 82 | Bacteroidia | NS5 marine group |
|  |  | Otu0023 | 79 | Gammaproteobacteria | OM60(NOR5) clade |
|  |  | Otu0132 | 79 | Gammaproteobacteria | OM60(NOR5) clade |
|  |  | Otu0022 | 78 | Verrucomicrobiae | *Roseibacillus* |
|  |  | Otu0049 | 78 | Bacteroidia | *Aurantivirga* |
|  |  | Otu0087 | 78 | Gammaproteobacteria | OM43 clade |
|  |  | Otu0138 | 78 | Gammaproteobacteria | SAR92 clade |
|  | Sterivex | Otu0233 | 32 | Gammaproteobacteria | Uncultured |
|  |  | Otu0011 | 29 | Acidimicrobiia | Candidatus *Actinomarina* |
|  |  | Otu0025 | 29 | Bacteroidia | Uncultured |
|  |  | Otu0074 | 29 | Gammaproteobacteria | SAR86 clade |
|  |  | Otu0075 | 29 | Gammaproteobacteria | SAR86 clade |
|  |  | Otu0146 | 29 | Gammaproteobacteria | SAR86 clade |
|  |  | Otu0005 | 25 | Alphaproteobacteria | Unclassified Rhodobacteraceae |
|  |  | Otu0006 | 25 | Cyanobacteria | *Prochlorococcus* MIT9313 |
|  |  | Otu0019 | 25 | Bacteroidia | NS5 marine group |
|  |  | Otu0050 | 25 | Bacteroidia | NS11-12 marine group |
| Thermaikos | Filter | Otu0037 | 3 | Acidimicrobiia | Candidatus *Actinomarina* |
|  |  | Otu0058 | 3 | Gammaproteobacteria | Unclassified Pseudomonadales |
|  |  | Otu0098 | 3 | Planctomycetes | *Blastopirellula* |
|  |  | Otu0106 | 3 | Alphaproteobacteria | Unclassified Rhodobacteraceae |
|  |  | Otu0112 | 3 | Actinobacteria | Candidatus *Aquiluna* |
|  |  | Otu0115 | 3 | Bacteroidia | *Winogradskyella* |
|  |  | Otu0216 | 3 | Bacteroidia | NS5 marine group |
|  |  | Otu0072 | 2 | Planctomycetes | Uncultured |
|  |  | Otu0079 | 2 | Alphaproteobacteria | Clade III |
|  |  | Otu0137 | 2 | Bacteroidia | *Aquibacter* |
|  | Gauze | Otu0134 | 7 | Verrucomicrobiae | *Roseibacillus* |
|  |  | Otu0267 | 7 | Bacteroidia | *Lewinella* |
|  |  | Otu0053 | 6 | Phycisphaerae | Urania-1B-19 marine sediment group |
|  |  | Otu0190 | 6 | Verrucomicrobiae | DEV007 |
|  |  | Otu0261 | 6 | Alphaproteobacteria | *Oceanicaulis* |
|  |  | Otu0177 | 5 | Verrucomicrobiae | *Roseibacillus* |
|  |  | Otu0287 | 5 | Alphaproteobacteria | Uncultured |
|  |  | Otu0315 | 5 | Gammaproteobacteria | *Legionella* |
|  |  | Otu0001 | 4 | Alphaproteobacteria | Clade Ia |
|  |  | Otu0010 | 3 | Alphaproteobacteria | Candidatus *Puniceispirillum* |
|  | Sterivex | Otu0190 | 31 | Verrucomicrobiae | DEV007 |
|  |  | Otu0241 | 25 | Cyanobacteria | *Synechococcus* CC9902 |
|  |  | Otu0017 | 24 | Cyanobacteria | *Cyanobium* PCC-6307 |
|  |  | Otu0033 | 24 | Rhodothermia | *Balneola* |
|  |  | Otu0058 | 23 | Gammaproteobacteria | Unclassified Pseudomonadales |
|  |  | Otu0112 | 23 | Actinobacteria | Candidatus *Aquiluna* |
|  |  | Otu0162 | 21 | Gammaproteobacteria | *Luminiphilus* |
|  |  | Otu0118 | 20 | Gammaproteobacteria | Uncultured |
|  |  | Otu0172 | 20 | Alphaproteobacteria | SAR116 clade |
|  |  | Otu0216 | 20 | Bacteroidia | NS5 marine group |
| **EUKARYOTES** | | | | | |
| **Location** | **Method** | **OTU** | **Connections** | **Class** | **Genus** |
| Kavala | Filter | Otu0136 | 2 | Sagenista | MAST-7 |
|  |  | Otu0063 | 2 | Mediophyceae | *Leptocylindrus* |
|  |  | Otu0004 | 1 | Urochordata | *Oikopleura* |
|  |  | Otu0006 | 1 | Syndiniales | Dino-Group-I Clade 1 |
|  |  | Otu0007 | 1 | Mediophyceae | *Leptocylindrus* |
|  |  | Otu0052 | 1 | Sagenista | MAST-7 |
|  |  | Otu0060 | 1 | Syndiniales | Dino-Group-II Clades 10 and 11 |
|  |  | Otu0070 | 1 | Mediophyceae | *Eucampia* |
|  |  | Otu0051 | 1 | Prymnesiophyceae | *Chrysochromulina* |
|  |  | Otu0159 | 1 | Dinophyceae | Unclassified Dinophyceae |
|  | Gauze | Otu0014 | 1 | Dinophyceae | *Pelagodinium* |
|  |  | Otu0085 | 1 | Spirotrichea | Strombidiidae |
|  | Sterivex | Otu0146 | 8 | Chrysophyceae | Chrysophyceae Clade EC2H |
|  |  | Otu0014 | 6 | Dinophyceae | *Pelagodinium* |
|  |  | Otu0185 | 5 | Filosa-Thecofilosea | *Protaspa* |
|  |  | Otu0293 | 5 | Spirotrichea | Strombidiidae |
|  |  | Otu0312 | 5 | Syndiniales | Dino-Group-II Clade 22 |
|  |  | Otu0063 | 4 | Mediophyceae | *Leptocylindrus* |
|  |  | Otu0103 | 4 | Picozoa | Picozoa |
|  |  | Otu0208 | 4 | Sagenista | *Aplanochytrium* |
|  |  | Otu0224 | 4 | Chytridiomycota | Spizellomycetales |
|  |  | Otu0001 | 3 | Dinophyceae | Unclassified Dinophyceae |
| Saronikos | Filter | Otu0024 | 4 | Arthropoda | *Oithona* |
|  |  | Otu0087 | 4 | Dinophyceae | Unclassified Gymnodiniaceae |
|  |  | Otu0117 | 4 | Picozoa | Picozoa |
|  |  | Otu0130 | 4 | Syndiniales | Dino-Group-III |
|  |  | Otu0169 | 4 | Gyrista | MAST-1C |
|  |  | Otu0061 | 3 | Syndiniales | Dino-Group-II Clade 10 and 11 |
|  |  | Otu0064 | 3 | Dinophyceae | Unclassified Dinophyceae |
|  |  | Otu0095 | 3 | Dinophyceae | *Torodinium* |
|  |  | Otu0176 | 3 | Dinophyceae | *Blastodinium* |
|  |  | Otu0010 | 2 | Arthropoda | *Paracalanus* |
|  | Gauze | Otu0054 | 9 | Prymnesiophyceae | *Phaeocystis* |
|  |  | Otu0007 | 8 | Mediophyceae | *Leptocylindrus* |
|  |  | Otu0049 | 8 | Bacillariophyceae | *Pseudo-nitzschia* |
|  |  | Otu0001 | 7 | Dinophyceae | Unclassified Dinophyceae |
|  |  | Otu0006 | 7 | Syndiniales | Dino-Group-I Clade 1 |
|  |  | Otu0117 | 6 | Picozoa | Picozoa |
|  |  | Otu0040 | 5 | Prymnesiophyceae | Unclassified Prymnesiaceae |
|  |  | Otu0084 | 5 | Unclassified | Unclassified |
|  |  | Otu0153 | 5 | Spirotrichea | Unclassified Oligotrichida |
|  |  | Otu0287 | 5 | Mediophyceae | *Chaetoceros* |
|  | Sterivex | Otu0038 | 3 | Acantharea | *Acanthometra* |
|  |  | Otu0011 | 2 | Dinophyceae | *Tripos* |
|  |  | Otu0160 | 2 | Chlorodendrophyceae | Unclassified Chlorodendraceae |
|  |  | Otu0022 | 2 | Dinophyceae | *Alexandrium* |
|  |  | Otu0169 | 2 | Gyrista | MAST-1C |
|  |  | Otu0001 | 1 | Dinophyceae | Unclassified Dinophyceae |
|  |  | Otu0005 | 1 | Cnidaria | Unclassified Hydrozoa |
|  |  | Otu0006 | 1 | Syndiniales | Dino-Group-I Clade 1 |
|  |  | Otu0014 | 1 | Dinophyceae | *Pelagodinium* |
|  |  | Otu0033 | 1 | Dinophyceae | *Warnowia* |
| Thermaikos | Filter | Otu0165 | 4 | Dinophyceae | *Protoperidinium* |
|  |  | Otu0015 | 3 | Dinophyceae | Unclassified Gymnodiniales |
|  |  | Otu0020 | 3 | Dinophyceae | *Gyrodinium* |
|  |  | Otu0013 | 2 | Cnidaria | Unclassified Hydrozoa |
|  |  | Otu0061 | 2 | Syndiniales | Dino Group-II Clades 10 and 11 |
|  |  | Otu0035 | 2 | Syndiniales | Dino Group-II Clades 10 and 11 |
|  |  | Otu0001 | 1 | Dinophyceae | Unclassified Dinophyceae |
|  |  | Otu0011 | 1 | Dinophyceae | *Tripos* |
|  |  | Otu0034 | 1 | Mamiellophyceae | *Bathycoccus* |
|  |  | Otu0050 | 1 | Filosa-Thecofilosea | Ebriida |
|  | Gauze | Otu0001 | 29 | Dinophyceae | Unclassified Dinophyceae |
|  |  | Otu0068 | 29 | Dinophyceae | Dinophyceae |
|  |  | Otu0039 | 27 | Mediophyceae | *Chaetoceros* |
|  |  | Otu0031 | 26 | Syndiniales | Dino-Group-III |
|  |  | Otu0104 | 26 | Cercozoa | Unclassified Cercozoa |
|  |  | Otu0088 | 24 | Spirotrichea | *Tintinnopsis* 13 |
|  |  | Otu0045 | 23 | Trebouxiophyceae | *Picochlorum* |
|  |  | Otu0058 | 23 | Nephroselmidophyceae | *Nephroselmis* |
|  |  | Otu0126 | 23 | Syndiniales | *Euduboscquella* |
|  |  | Otu0203 | 23 | Choanoflagellatea | Monosigidae Group B |
|  | Sterivex | Otu0069 | 17 | Syndiniales | Dino-Group-II Clade 44 |
|  |  | Otu0040 | 16 | Prymnesiophyceae | Unclassified Prymnesiaceae |
|  |  | Otu0051 | 16 | Prymnesiophyceae | *Chrysochromulina* |
|  |  | Otu0112 | 15 | Picozoa | Picozoa |
|  |  | Otu0039 | 13 | Mediophyceae | *Chaetoceros* |
|  |  | Otu0199 | 13 | Pterocystida | Pterocystida |
|  |  | Otu0030 | 12 | Dinophyceae | *Gymnodinium* |
|  |  | Otu0031 | 12 | Syndiniales | Dino-Group-III |
|  |  | Otu0073 | 12 | Sagenista | MAST-4A |
|  |  | Otu0023 | 11 | Prymnesiophyceae | *Chrysochromulina* |

Figure S2 Rarefaction curves for bacterial communities, showing observed OTU richness as a function of sequencing depth. Curves are grouped by sampling method and location

Figure S3 Rarefaction curves for eukaryotic communities, showing observed OTU richness as a function of sequencing depth. Curves are grouped by sampling method and location.
